# Oxygen nanobubbles revert hypoxia by methylation programming

**DOI:** 10.1038/s41598-017-08988-7

**Published:** 2017-08-24

**Authors:** Pushpak N. Bhandari, Yi Cui, Bennett D. Elzey, Craig J. Goergen, Christopher M. Long, Joseph Irudayaraj

**Affiliations:** 1 Department of Agricultural and Biological Engineering, Bindley Bioscience Center, Purdue Center for Cancer Research, Purdue University, 225 South University Street, West Lafayette, Indiana, 47907 USA; 2 Department of Comparative Pathobiology, Purdue University, West Lafayette, Indiana, 47907 USA; 3 Weldon School of Biomedical Engineering, Purdue University, West Lafayette, Indiana, 47907 USA; 4 Purdue University Center for Cancer Research, West Lafayette, Indiana, 47907 USA

## Abstract

Targeting the hypoxic tumor microenvironment has a broad impact in cancer epigenetics and therapeutics. Oxygen encapsulated nanosize carboxymethyl cellulosic nanobubbles were developed for mitigating the hypoxic regions of tumors to weaken the hypoxia-driven pathways and inhibit tumor growth. We show that 5-methylcytosine (5mC) hypomethylation in hypoxic regions of a tumor can be reverted to enhance cancer treatment by epigenetic regulation, using oxygen nanobubbles in the sub-100 nm size range, both, *in vitro* and *in vivo*. Oxygen nanobubbles were effective in significantly delaying tumor progression and improving survival rates in mice models. Further, significant hypermethylation was observed in promoter DNA region of BRCA1 due to oxygen nanobubble (ONB) treatment. The nanobubbles can also reprogram several hypoxia associated and tumor suppressor genes such as MAT2A and PDK-1, in addition to serving as an ultrasound contrast agent. Our approach to develop nanosized oxygen encapsulated bubbles as an ultrasound contrast agent for methylation reversal is expected to have a significant impact in epigenetic programming and to serve as an adjuvant to cancer treatment.

## Introduction

Epigenetics plays an important role in regulating the expression of genes and corresponding cellular and molecular pathways^1^. DNA methylation (*i.e*. covalent addition of a methyl group to the C-5 carbon of the cytosine group in DNA) constitutes an important step in epigenetic programming and has been implicated in gene expression^2–4^. Addition of methyl groups to the cytosine derivatives in the DNA sequence can render the associated genes transcriptionally inactive^5^. DNA demethylation can lead to a hypomethylated state, but is counteracted by active DNA methylation to achieve a balanced methylation level^6, 7^. In neoplasia, the unregulated proliferation of cellular mass, without a sustainable rate of angiogenesis, leads to the development of hypoxic conditions inside the tumor^8^. In response to the pervasive hypoxic environment, several oncogenic processes occur in the cells, one of which is epigenetic alterations, resulting in an increase in tumor growth and survival of cancer cells^9, 10^. These alterations include global hypomethylation (primarily of oncogenes rendering them active)^11^, gene-specific hypermethylation (of CpG islands in the promoter regions of tumor suppressor genes, rendering them inactive), and inducing cell proliferation via dysregulated cell growth^3^. Ten-eleven translocation (TET) enzymes are a group of Fe^2+^ and α-ketoglutarate dependent dioxygenases that oxidize the conversion of 5mC to 5hmC and other downstream derivatives^12–14^. In mammalian cells, TET enzyme is the only characterized factor mediating the active DNA demethylation process^12, 13^. The activity of TET enzymes that have been shown to catalyze DNA demethylation is also limited by oxygen supply^14^. Although epigenetic therapy in the laboratory and clinics have largely focused on changes at gene promoters^15, 16^, epigenetic abnormalities such as DNA 5mC methylation across the genome^17^ are now being looked upon as diagnostic (tumor staging, outcome prediction, and malignancy) and therapeutic targets (epigenetic drugs). Regulation of the hypoxic microenvironment and epigenetic events are promising steps in anticancer therapies because several hypoxia^18–20^ and epigenetic^15, 21–23^ targeted therapies have shown efficacy in the clinic^24–26^. Further, this view is also supported by findings on the effect of supplemental oxygen that weaken the hypoxia-driven pathways to improve cancer immunotherapy to promote tumor regression^27^.

Supplemental respiratory oxygen has shown significant lung tumor regression and long-term survival in mice and is being proposed as a treatment option^27–29^ by combining it with existing cancer immunotherapies. However, the toxicity and nonspecific inflammatory response^30, 31^ in addition to its large instrumentation footprint, diminishes its potential as a viable therapeutic option. Hence the motivation to develop injectable and safer treatment options that weaken the hypoxia-driven global hypomethylation and hypoxia-adaptive pathways. Herein, we reason that delivery of nanosize oxygen bubbles specifically to hypoxic regions would help to regulate the epigenetic state by destabilizing the hypoxia-mediated pathways such as hypoxia inducible factor (HIF)^32^ that promote tumor progression. Global 5mC methylation along with HIF-1α levels were monitored *in vitro* and *in vivo* during the course of tumor regression upon hypoxia reversal, using human cervical cancer (HeLa) and murine bladder cancer (MB49) tumor models. Further, promoter methylation analysis was used to assess a group of tumor suppressor genes. Our results indicate that the oxygen nanobubbles can potently alter the epigenetic state of the cell cycle-related genes and mitigate cancer cell proliferation. Our approach provides an injectable, nano-scale oxygen delivery platform to mitigate hypoxia and to alter the epigenetic state, thus providing an opportunity for epigenetic therapy approaches by destabilizing the hypoxia-adaptive pathways in the tumor.

## Results

**Figure 1:**
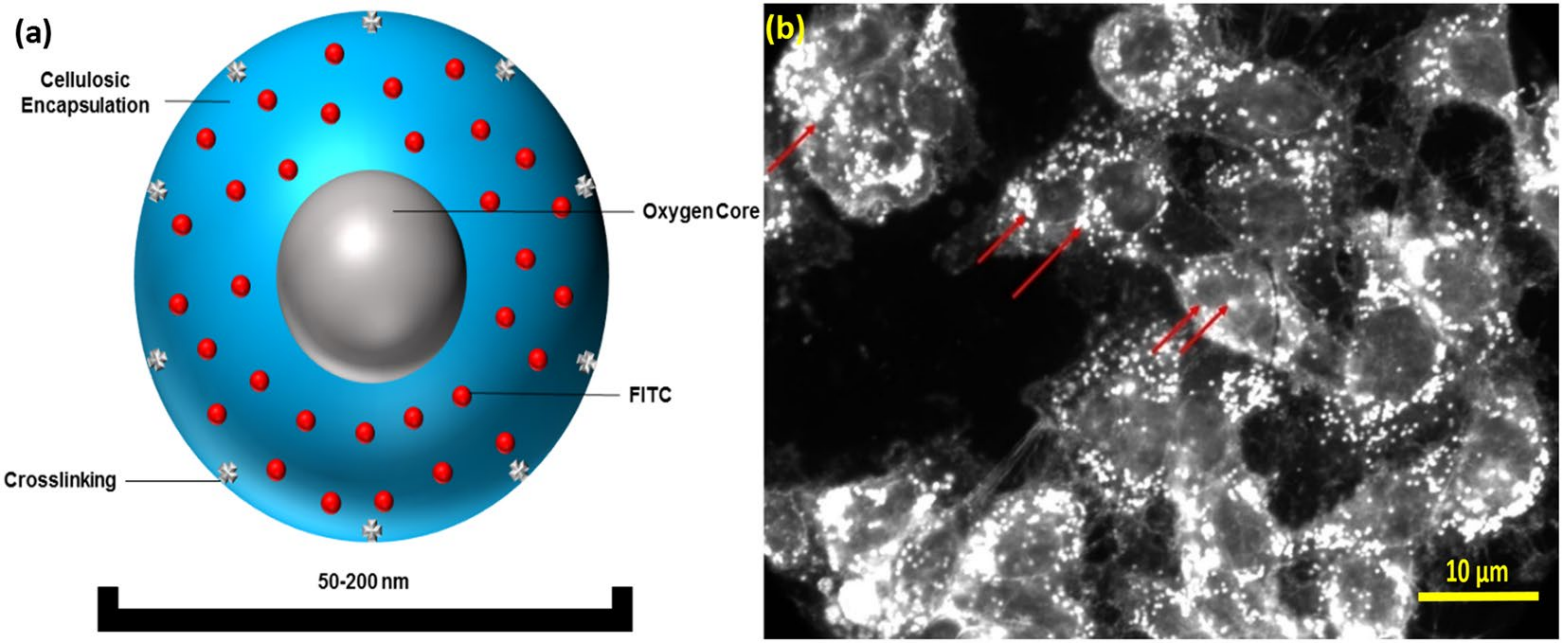
Oxygen Nanobubble configuration and mechanism of 5mC hypermethylation. (**a**) Schematic representation of oxygen nanobubble; (**b**) Oxygen nanobubbles (red arrows) localize within HeLa cells in the cytoplasm as well as the nucleus. Significantly enhanced dark field microscopy images are provided. Scale bar = 10 µm.

**Figure 2:**
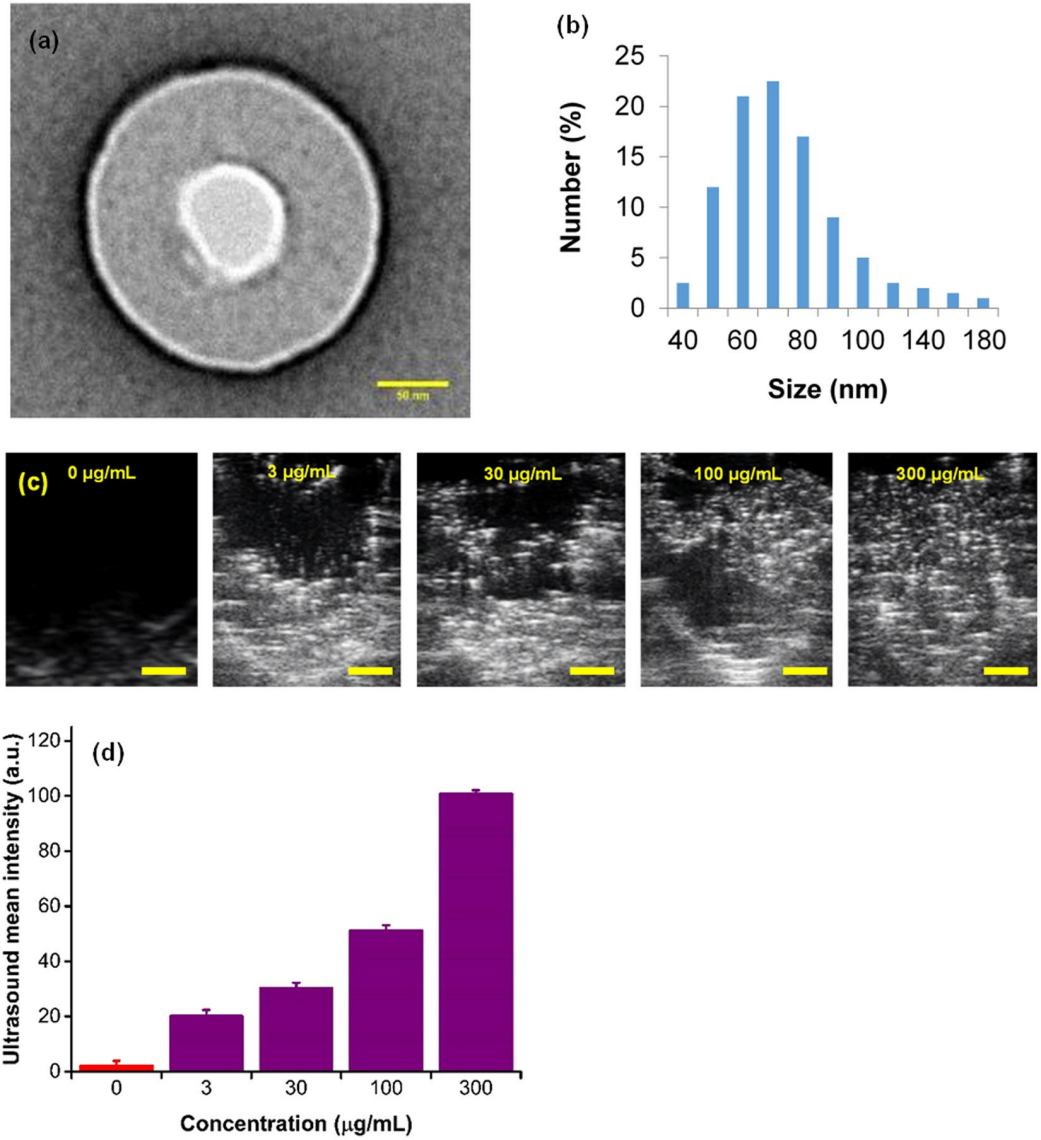
*In vitro* characterization, hypoxia reprogramming, and imaging of nanobubbles. (**a**) TEM image of nanobubble with an oxygen compartment at the core surrounded by sodium carboxymethylcellulose shell. Scale bar = 50 nm. (**b**) Dynamic light scattering (DLS) size distribution of nanobubbles. (**c**) Ultrasound images of the corresponding signal generated from varying concentrations of nanobubbles (0–300 µg/mL). The contrast generated is due to oxygen trapped inside nanobubbles. Scale bar = 1 mm. (**d**) Graph displaying averaged mean grey scale intensity corresponding to increasing concentrations of nanobubbles (0–300 µg/mL). The results are mean values from three independent experiments. Error bars represent ± s.d. Note that there is a significant linear relationship between mean ultrasound gray scale intensity and concentration of nanobubbles (see Supplementary Fig. 1).

We hypothesize that alteration of DNA hypomethylation in hypoxic cancer cells can be achieved by the delivery of oxygen to the cellular microenvironment with nanosize oxygen bubbles (Fig. 1a,b). In particular, our approach consists of encapsulating oxygen inside a sodium carboxymethylcellulose polymeric shell (Fig. 1a) to form nanobubbles 100–200 nm in diameter (Fig. 2a,b) by a crosslinking step^33^. High resolution TEM micrographs show that the synthesized nanobubbles have a spherical shape (Fig. 2a) and contain an oxygen core at the center and a ~50 nm carboxymethyl cellulose shell encapsulating the nanobubble. Dynamic light scattering (DLS) shows that the size distribution of nanobubbles is in the range between 50–200 nm with a normal distribution centered around 70 nm (Fig. 2b). Further, sodium carboxymethylcellulose is a commercially used, FDA-approved pharmaceutical excipient. Upon uptake, the acidic microenvironment around and inside the tumor cells^34^ will cause the nanobubble shells to disintegrate, thereby increasing the cellular oxygen levels. We expect the release of oxygen inside the hypoxic cells will destabilize the hypoxia-adaptive pathways and reprogram the cellular epigenome to attain normal DNA methylation levels, or cause global hypermethylation. The targeted oxygen delivery is also expected to promote the regression of tumor growth in the hypoxic xenografted MB49 (bladder cancer) and HeLa (cervical cancer) tumors.

In addition to reoxygenation, we anticipate that oxygen nanobubbles will act as contrast agents for ultrasound imaging. Nanobubbles also possess unique light scattering and absorption characteristics as demonstrated using dark field microscopy (Fig. 1b) and in our prior work^33^. To test the ultrasound imaging intensity response to increasing concentration of nanobubbles, agarose gel molds were prepared with varying concentration of nanobubbles (Supplementary Fig. 1). B-mode ultrasound images of injected nanobubbles are shown in Fig. 2c and the corresponding mean gray scale intensity measurements are depicted in Fig. 2d. A linear increase in ultrasound grey scale imaging intensity (Fig. 2d and Supplementary Fig. 2) was observed as a function of nanobubble concentration (R^2^ = 0.95) in the concentration range evaluated (0–300 µg/mL). Further, HeLa cell cultures grown in tissue culture plates were incubated either with oxygen nanobubbles or phosphate buffer saline (PBS). After incubation for 24 h, the cell cultures were imaged using a 256-element 22–55 MHz ultrasound transducer with a center frequency of 40 MHz (for sample preparation and imaging details, see Methods Section). Images show that the spherical nanobubbles are suspended in the media as well as around the HeLa cells adhered to the bottom of the plate (Supplementary Fig. 3b) compared to the HeLa cell culture without nanobubbles (Supplementary Fig. 3a). The ultrasound gray scale imaging intensity in cell cultures with nanobubbles was significantly higher than the control without the addition of nanobubbles (Supplementary Fig. 4). The proposed design allows for customization of its size to accommodate various oxygen carrying capacity capable of generating different ultrasound contrast intensity.

**Figure 3:**
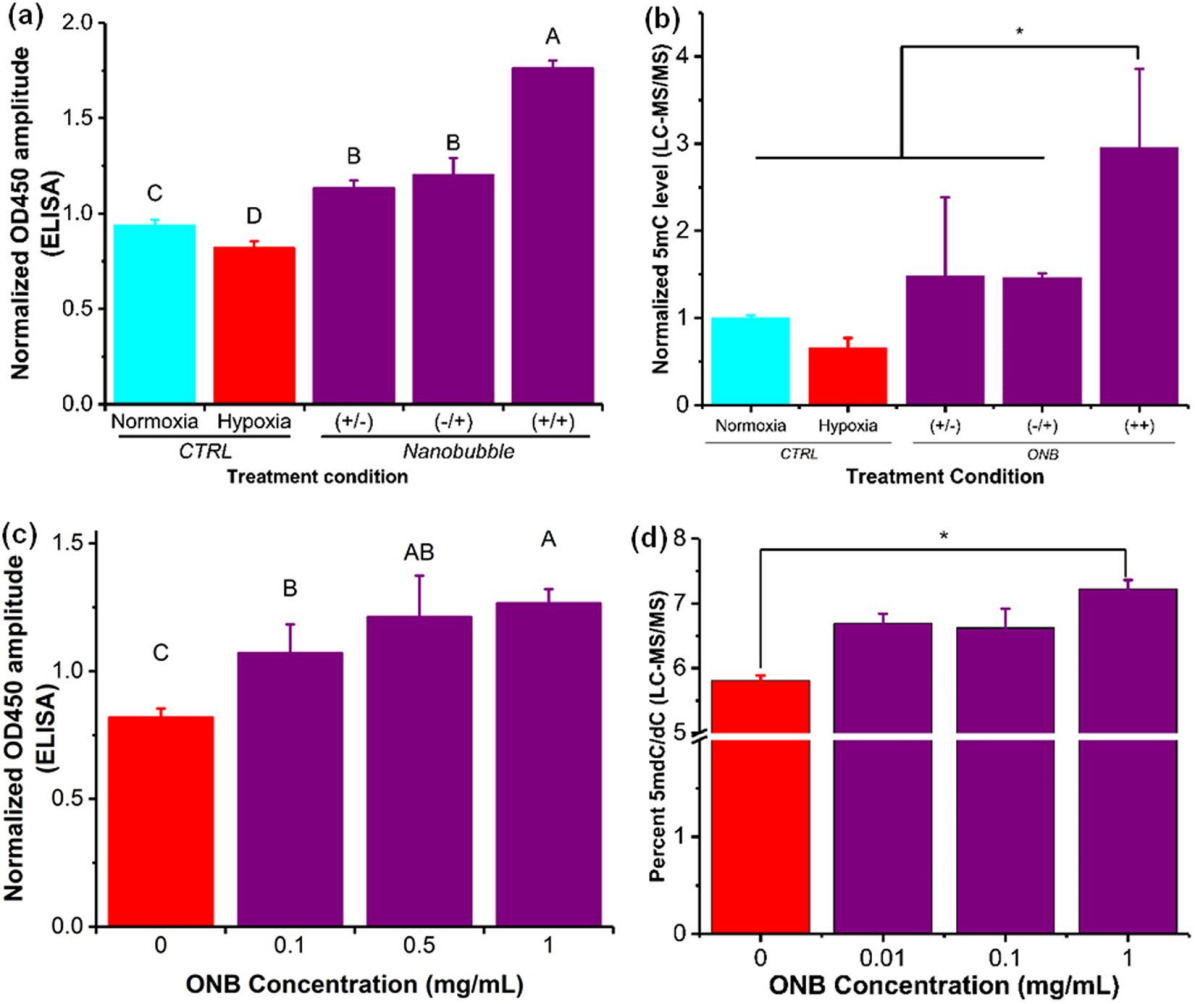
ONBs perturb 5mC hypomethylation *in vitro*. (**a**) 5mC methylation levels in HeLa cells as measured using ELISA for varying treatments, and 0.5 mg/mL nanobubble concentration, to identify the relation between treatment frequency and the total time of incubation. (+/−) Signifies samples with addition of nanobubbles 0 hours after incubation and no addition of nanobubbles after 24 hours of incubation. (−/+) Signifies the samples with no addition of nanobubbles after 0 hours of incubation but addition of nanobubbles after 24 hours of incubation. (+/+) Signifies samples with the addition of nanobubbles after both 0 hours and 24 hours of incubation. (**b**) 5mC methylation levels as measured using ELISA for varying concentration of nanobubble treatments under hypoxia for HeLa cells. The nanobubble treatment volume and time of treatment was the same for all samples. (**c**) Normalized 5mC methylation levels in HeLa cells as measured using LC-MS/MS for varying treatments, and 0.5 mg/mL nanobubble concentration, to identify the relation between treatment frequency and the total time of incubation. (+/−) Signifies samples with addition of nanobubbles 0 hours after incubation and no addition of nanobubbles after 24 hours of incubation. (−/+) Signifies the samples with no addition of nanobubbles after 0 hours of incubation but addition of nanobubbles after 24 hours of incubation. (+/+) Signifies samples with the addition of nanobubbles after both 0 hours and 24 hours of incubation. Samples were analyzed using LC-MS/MS. (**d**) 5mC methylation levels in HeLa cells as measured using LC-MS/MS for varying concentration of nanobubble treatments under hypoxia for HeLa cells. The nanobubble treatment volume and time of treatment was the same for all samples. The results are mean values from three independent experiments ± s.d. Mean values not connected by same letter are significantly different. Significance established with one-way analysis of variance (ANOVA) with Tukey’s multiple comparison test. *P < 0.05.

The 5mC levels in the hypoxic regions have been shown to rapidly decrease, independent of the cell proliferation cycle^35^. In our experiments, DNA was extracted after 48 h of incubation following a factorial experiment design (data not shown) to ensure sufficient time for the methylation changes to take effect^35^. Colorimetric enzyme-linked immunosorbent assay (ELISA) was used to quantify 5mC levels^36^ after the exposure of nanobubbles to a hypoxic environment (Fig. 3a,c) and the 5mC levels were further validated using liquid chromatography-mass spectrometry (LC-MS/MS) (Fig. 3b,d). The DNA methylation levels measured from cells exposed to ONBs for different time periods (Fig. 3a,b) showed a distinct decrease (α = 0.05) in the DNA methylation levels in hypoxic cells compared to the control. Further, irrespective of the time of dose (start of treatment at 0 h or 24 h), no significant difference was observed in the methylation levels (Fig. 3a,b and Supplementary Fig. 6). However, in cells treated with nanobubbles, i.e. addition of nanobubbles (0.5 mg/mL) at 0 h and after 24 h of incubation, a rapid and significant increase in 5mC DNA methylation levels was observed. Under hypoxia, DNA 5mC levels (measured as OD450 absorbance) linearly increased (*P* < 0.004, R^2^ = 0.58) corresponding to an increase in oxygen nanobubble concentration (Fig. 3c,d and Supplementary Fig. 5). A significant difference was observed (Fig. 3c,d) between 0, 0.1, and 1 mg/mL of nanobubble concentration (α = 0.05). The trends for different treatment conditions and oxygen nanobubble concentrations were similar and validated by ELISA and LC-MS/MS. Our observations infer that active 5mC levels in hypoxic tumor cells can be increased using oxygen nanobubbles in a dose-dependent manner, *in vitro*.

**Figure 4:**
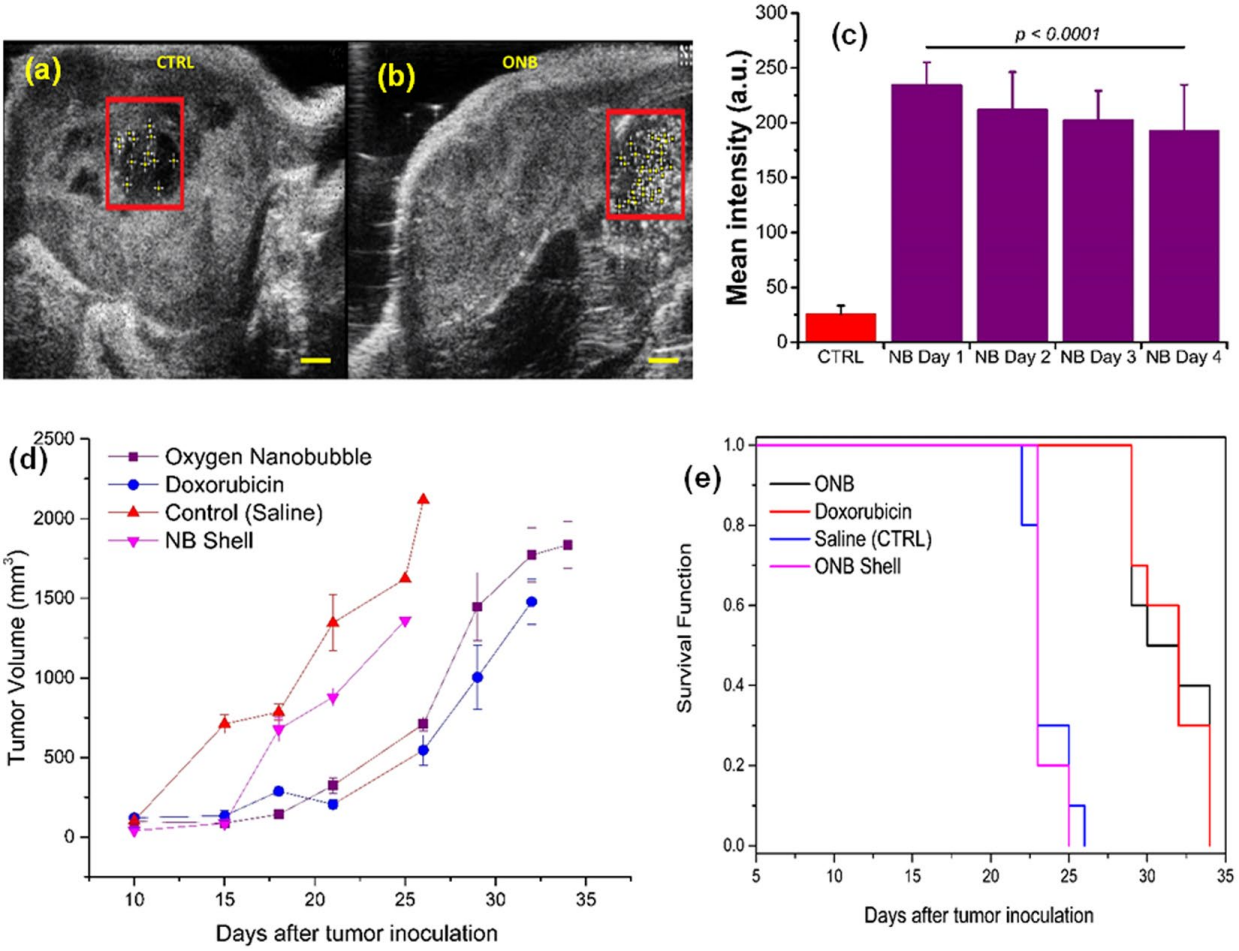
Monitoring *in vivo* biodistribution and tumor mitigating ability of oxygen nanobubbles in hypoxic cells. (**a**,**b**) Representative ultrasound B-mode images acquired using ultrasound transducer of NSG mice infused intratumor with 100 µL nanobubbles (**a**) and saline (**b**). Boxed outline shows region of interest (ROI) positioned manually at the center of injections and used to quantify ultrasound signal. Scale bar = 1 mm (**c**) Mean ultrasound gray scale intensity from ROIs covering saline-injected (CTRL) and nanobubbles-injected (red bars, over 4 days) tumor. Results are mean ± s.d. for three independent measurements for each group. (**d**) Tumor volume of MB49-tumor bearing mice treated with oxygen nanobubble (1 mg/mL ONB, 0.1 µg/kg mouse weight), doxorubicin (40 mg/kg mouse weight), saline (control), or nanobubble shell (1 mg/mL, 0.1 µg/kg mouse weight). The treatment was administered intratumorally into the mouse on day 12 when the tumors reached 10 mm ± 2 mm (n = 10 for doxorubicin, control and ONB groups; n = 5 for NB shell group). (**e**) Surviving fractions of tumor-bearing mice. Mean survival times in days were calculated for each group.

Finally, to demonstrate 5mC hypomethylation reversal by nanobubbles *in vivo*, we performed intratumoral injection of oxygen nanobubbles into severely hypoxic MB49 and HeLa tumors (four weeks post-xenografting). 3D volumetric measurements were also performed using a linear step motor with 3D mode in the ultrasound imaging system. Images indicate that nanobubbles successfully enhanced the imaging contrast (Fig. 4a) and localized subcutaneously. Ultrasound B-mode images indicate that the oxygen nanobubbles are localized within the tumors and generate a significant increase in ultrasound contrast intensity (Fig. 4a) whereas the control mice injected with saline (Fig. 4b) did not show any significant ultrasound contrast intensity. The ultrasound contrast intensity inside tumors injected with nanobubbles decreased over four days of monitoring (Fig. 4c) indicating that the acidic pH microenvironment of hypoxic tumors could contribute to the disintegration of the nanobubbles to facilitate the diffusion of the encapsulated oxygen gas into the surrounding microenvironment^37^. Oxygen concentrations inside the tumor increased by around 140% after the injection of ONBs (Supplementary Fig. 7). The oxygen concentrations decreased to the original hypoxic levels (~50 mmHg) 5 days post-injection indicating that the tumor remained oxygenated for around 5 days post-injection. Further, the unregulated proliferation of cells and angiogenesis effect are hypothesized to overcome the oxygenation and return the cell to its hypoxic state.

The *in vivo* anti-tumor efficacy of nanobubbles was evaluated in MB49-tumor-bearing mice (Fig. 3d,e; n = 10). Compared to control (saline injection), doxorubicin had a significant effect as expected (*P* < 0.01) in slowing tumor growth (Fig. 3d). Oxygen nanobubbles also showed a significant (oxygen nanobubbles vs. saline *P* < 0.01) decrease in tumor growth (Fig. 3d, Supplementary Fig. 8). Further, Kaplan-Meier survival rate analysis (Fig. 3e) showed that doxorubicin and nanobubble groups have a significantly higher (*P* < 0.0001) survival rate (mean survival of 31.5 days) than the untreated group (mean survival of 23.5 days) and the group treated with nanobubble shell (mean survival of 23.4 days). No significant difference was observed between nanobubble shell and control (saline) indicating that the therapeutic effect was due to oxygen encapsulated in the nanobubble. Further, no significant differences were observed in the overall survival rate between the nanobubble and doxorubicin groups (mean survival of 31.5 days for both) highlighting the potential of nanobubbles in treating solid tumors. We observed that doxorubicin administered mice had significant toxicity as observed by the difference in mouse weight (Supplementary Fig. 9). Mice in doxorubicin treated group had 15–20% lower weight at the end of the study compared to mice in control group consistent with previous documented studies on doxorubicin toxicity^38–44^. Further, no adverse toxicity events (mortality, convulsions, lethargy, or coma)^45^ were noted with the nanobubble group whereas doxorubicin groups showed significant toxicity, as previously noted in the literature^40, 41, 43, 46^. The heart rate and breathing were monitored in animals and found to be normal for the ONB group. Mice treated with doxorubicin registered three toxicity events (lethargic mice, ulcerated tumors, and significant weight loss) that required euthanasia. Further, ulcerated tumors were commonly noticed in the doxorubicin treated mice^39, 40, 42^. To account for any potential toxicity to the surrounding normal tissue, 100 µL of oxygen nanobubbles (1 mg/mL) was injected intramuscularly in the region around the tumor in MB49 tumor bearing mice (n = 5). No significant growth or necrosis in the muscle was observed for over 28 days (Supplementary Fig. 10) indicating that the nanobubbles are not toxic to normal tissue and appear to have the required function in the hypoxic regions of the tumor.

**Figure 5:**
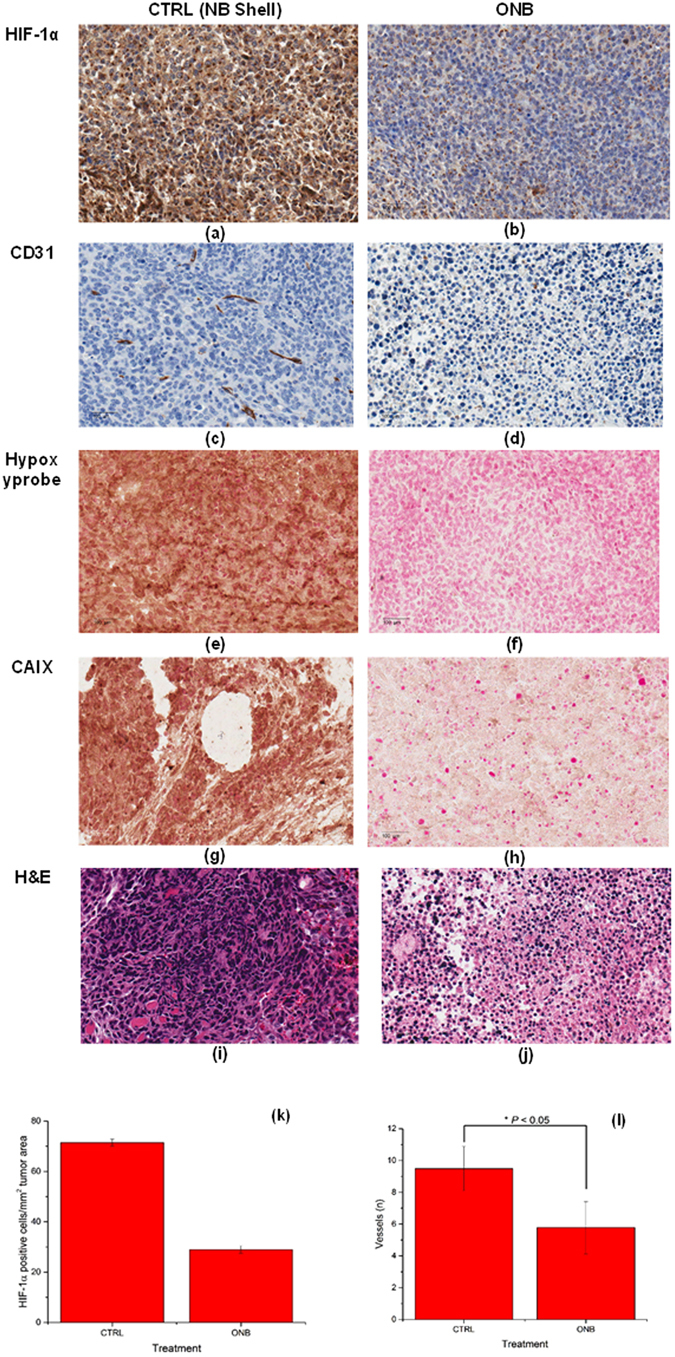
Immunohistochemistry staining pattern of metabolic (CD31, CAIX), hypoxic (HIF-1α, Hypoxyprobe), and tissue morphology markers. Microscopic images of MB49 tumor tissues treated with nanobubble shell (CTRL) or oxygen nanobubbles (Treatment): Expression of HIF-1 (immunohistochemistry, magnification ×40, scale bar = 100 µm) in CTRL (**a**) and ONB (**b**) treated tumors shows strong HIF-1 expression in control tumors and weak positive expression in ONB treated tumors. Representative IHC images for control (**c**) and ONB (**d**) treated tumor tissues show that CD31 expression levels were significantly lower in ONB treated groups compared to control (*P* < 0.05). Representative images of hypoxia immunostaining for Hypoxyprobe-1 adducts and tissue necrosis in tumor sections for control (**e**) and ONB-treated (**f**). Representative images of CAIX staining in tumor sections for control (**g**) and ONB-treated (**h**). H&E-stained histology images of the corresponding tumor sections showing significantly lower tumor cell volume (% cell volume/mm^2^ of tumor mass) for mice treated with ONB (**j**) compared to sections of tumors injected with ONB shell (**i**). Images captured at ×40 objective magnification. Scale bar = 100 µm. HIF-1 protein localization analysis by immunohistochemistry showing significant reduction in the number of HIF-1 positive cells per mm^2^ of tumor area with ONB treatment (**k**). Histomorphometric quantification of CD31 vessel density (**l**).

Mitigation of chronic hypoxia due to oxygen nanobubbles *in vivo* was further characterized by quantifying and visualizing the expression of endogenous and hypoxia markers in MB49 tumors using established protocols^47–49^. Consistent with *in vitro* results, we found a decrease in the expression of HIF-1 in the IHC imaging upon treatment with ONB (Fig. 5a,b). Further, HIF-1 protein localization analysis by immunohistochemistry shows significant reduction in the number of HIF-1 positive cells per mm^2^ tumor area with ONB treatment (Fig. 5k). CD31 angiogenesis assay was performed on control (Fig. 5c) and ONB-treated (Fig. 5d) tumor sections and analyzed histomorphometrically^48, 49^. The number of microvessels in sections stained with CD31 were counted to evaluate the anti-angiogenesis effect of ONB. ONB was significant in reducing the microvessel density compared to control (Fig. 5l), which may explain the changes in tumor vascularization due to ONBs. Since enhanced oxygenation suppresses the growth of tumor cells, there is no demand for high blood supply. Moreover, some inflammatory factors released along with cell apoptosis may inhibit angiogenesis. This finding is consistent with recent reports by Kammertoens *et al*. (Nature, 2017)^50^ which state that IFNγ-induced tumor ischaemia results in an arrest of blood flow and regression in tumor size and blood vessels. Significantly lower amounts of hypoxia were observed in tumors treated with ONB compared to control tumors (P < 0.05) and our results (Fig. 5e,f) were consistent with previous reports on hypoxia targeting^47^. To confirm our findings, CAIX expression was visualized using CAIX IHC (Fig. 5g,h). CAIX expression decreased significantly upon ONB treatment. Thus, strong correlation of CAIX with HIF-1, pimonidazole (Hypoxyprobe), and CD31 staining was observed as reported by Rademakers *et al*.^47^. We also observed that tumor cell volume (% cell volume/mm^2^ of tumor mass) was significantly lower for mice treated with ONB (Fig. 5i,j). Large regions of cell death were observed in the ONB-treated group, which demonstrated a higher anti-tumor activity, consistent with the results of tumor growth inhibition. In conclusion, different staining presented here are crucial to elucidate the response of hypoxic tumor cells to the oxygen nanobubbles and also extends previous observations of supplemental oxygen in enabling tumor regression by decreasing intratumoral hypoxia and concentrations of extracellular adenosine^27, 51^. No cytotoxic effects of the nanobubbles were observed in any of the organs analyzed (Supplementary Fig. 11).

**Figure 6:**
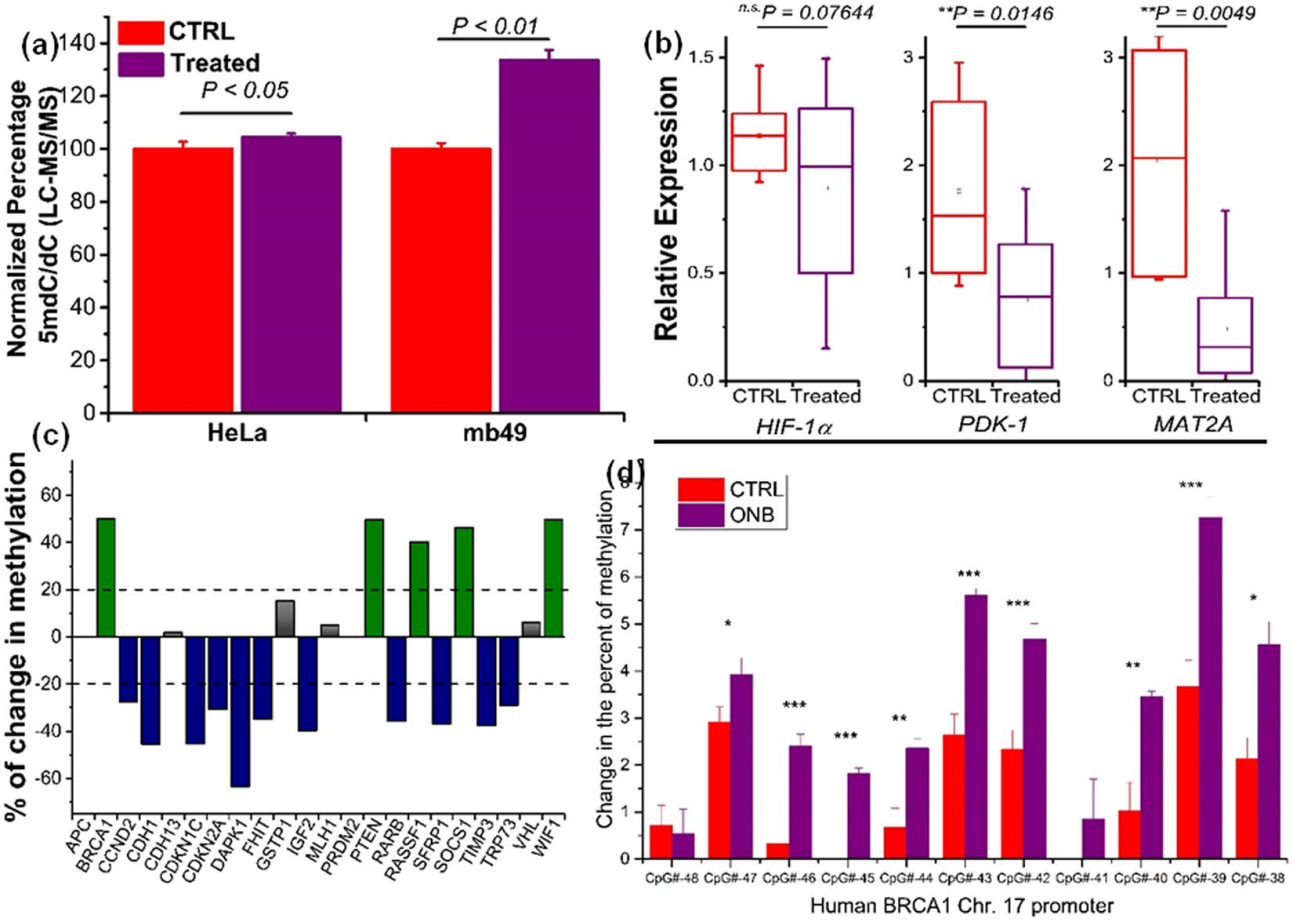
Quantitative real-time PCR, LC-MS, and gene-specific methylation analysis of *in vivo* tumors. (**a**) LC - MS/MS quantitation of 5mC levels in terms of ratios of 5-methyl-2′-deoxycytidine (5mdC) to that of deoxycytidine (dC) in nanobubble shell (CTRL) and oxygen nanobubble (Treatment) treated groups for HeLa (left) and MB49 (right) cells in mice models. (**b**) Transcription levels of HIF-1α, PDK-1, and MAT2A determined by qRT-PCR. (n = 16, *p-value* was calculated by ANOVA). (**c**) Changes in promoter methylation of the 22 selected tumor suppressor genes. (**d**) Oxygen nanobubble induced changes in DNA methylation at the CpG islands of Human BRCA1 promoter region. The extent of methylation was compared between *in vivo* tumors treated with either nanobubbles or saline. Methylation levels were measured by bisulfite treatment of the DNA followed by pyrosequencing and expressed as % change at the promoter region of chromosome 17. ***P < 0.001, **P < 0.01, *P < 0.05.

In addition to the EIA quantification of HeLa cells *in vitro*, liquid chromatography-tandem mass spectrometry (LC-MS/MS) analysis was used to further validate our observation of an increase of 5mC upon treatment with nanobubbles. We determined that the 5mdC/dC ratios are significantly higher in HeLa (*P* < 0.05) and MB49 (*P* < 0.01) than those in control (Fig. 6a). Further, qRT-PCR was performed to observe the transcriptional reversion of HIF-1α, PDK-1, and MAT2A upon nanobubble treatment (Supplementary Fig. 12). As shown in Fig. 6b, both PDK-1 and MAT2A targets were generally down-regulated in the nanobubble-treated tumor tissues compared to controls whereas no significant effect was observed for HIF-1α. MAT2A maintains the DNA methylation homeostasis by regulating the metabolism of S-adenosylmethionine (SAM)^52^. Recently, it has been found that the expression of MAT2A is HIF-1α-dependent and would be concurrently activated under hypoxia to induce global DNA hypomethylation in liver cancer^53^. Moreover, the expression level of PDK-1 and MAT2A was found to be negatively correlated with the tumor size and metastasis. The set of experiments conducted provides evidence of the therapeutic potential of oxygen nanobubbles by rescuing the global DNA hypomethylation in hypoxic environments.

We profiled a panel of 22 tumor suppressor genes for their promoter methylation status. Over half of the assessed genes harbor DNA hypermethylation (Supplementary Fig. 13), indicating their relevant deregulation in the xenografted tumors. Interestingly, upon nanobubble treatment, 11 genes experienced a significant decrease in promoter methylation (Fig. 6c). This observation suggests that reversion of hypoxia by nanobubble treatment has the potential to reshape the cancerous landscape of DNA methylation to a relatively normal status, *i.e*., remethylation at the global level and demethylation at specific tumor suppressor genes. An important function reactivated under nanobubble treatment is the cell cycle controlling mechanism mediated by p57 (CDKN1C), p21 (CDKN2A), and p73 (TRP73). These genes are potent regulators to suppress abnormal cell proliferation and to induce apoptosis in cancers^54, 55^. More importantly, aberrant hypermethylation was found to silence their functions in a number of cancers^56–58^. Hence, epigenetic regulation of these genes could be partly responsible for the inhibition of tumor progression observed in our *in vivo* experiments. Further details of the key genes involved and pathways are provided in Supplementary Fig. 14.

To validate the qRT-PCR results, pyrosequencing was performed on the bisulfite-treated DNA extracted from *in vivo* tumor samples. The promoter DNA region for BRCA1 was studied based on available DNA methylome profiles from previous work^59^. Most of the assayed gene regions displayed significant hypermethylation in ONB treated groups compared to control (Fig. 6d). The mean percent methylation for the entire region studied was highly significant for the ONB treated groups compared to control (*P* < 0.0001) validating our RT-PCR results (Fig. 6c). Nine of the eleven CpGs analyzed showed significant increase (*P* < 0.05) in methylation due to ONB treatment compared to control (Fig. 6d). Hypoxia mediated 5hmC loss and 5mC gain in promoter regions has been observed previously by Thienpont *et al*.^14^. The methylation levels observed for the extended promoter region studied were consistent with those published in literature^59^.

Our study provides the first evidence of the therapeutic potential of nanosize oxygen bubbles in reverting the methylation status of hypoxic tumor regions. No acute toxicity was observed in mice due to nanobubble accumulation. The heart rate and breathing were monitored in animals and were found to be normal. In summary, our results from mouse models show that oxygen nanobubbles are effective in reverting the methylation state and we expect our methods to be foundational in on-target ultrasound guided cargo delivery and as an adjuvant.

## Discussion

Our findings reveal that it is possible to induce epigenetic changes (global 5mC DNA methylation) in hypoxic neoplasia cells by treating tumors with oxygen nanobubbles. From our preliminary work, we found that reversal of hypoxic tumor microenvironment using ONB’s would mitigate global DNA hypomethylation for epigenome stabilization and reactivate a host of tumor suppressor genes to inhibit abnormal cell proliferation^60–63^. Epigenetic regulation is crucial for gene expression, one of the key determinants of cellular fate^1^. DNA methylation and histone modification are some of the dominant epigenetic modifications at play in regulating gene expression. Tumor hypoxia results from inadequate blood flow in tissues, reduced diffusion of micronutrients due to tumor expansion, and reduced O_2_ carrying capacity of blood^8^. In order to survive hypoxic conditions, the cells undergo epigenetic regulation involving global hypomethylation and targeted hypermethylation of key genes^11^ among other cellular functions.

Our study is also the first to report on the synthesis of oxygen nanobubbles less than 100 nm in size (Fig. 2a,b). We develop our delivery vehicle using sodium carboxymethyl cellulose (NaCMC), an FDA-approved pharmaceutical excipient, to encapsulate oxygen along with fluorophore, when needed by cross-linking. The nanobubble is non-cytotoxic and can easily localize in the tumor region due to its size. In addition to the reversal of 5mC DNA methylation levels, the oxygen nanobubbles offer the capability to arrest tumor progression due to the supplemental oxygen being delivered directly to tumor cells. Further, the echogenic and fluorescing properties of nanobubbles will permit the bubbles to be tracked as they diffuse through the target environment/organism and localize in specific regions to deliver oxygen. The importance of our results is underscored by the antitumor effects of hyperoxia on pulmonary MCA205 tumors in mice housed in 60% O_2_ breathing chambers. Hatfield *et al*.^27, 51^ demonstrated that this effect was largely due to the inhibition of the hypoxic immunosuppressive tumor microenvironment which permits successful adaptive T cell therapy. Although local application of ultrasound is an available option for enhanced delivery as demonstrated in literature^64–68^, our proof-of-concept work did not utilize any localized or focused triggering ultrasound.

Although no significant toxicity was observed due to administration of nanobubbles, the epigenetic and cellular responses of normal cells to oxygen nanobubbles and supplemental oxygen are yet to be completely established. Future work could involve encapsulation of epigenetic drugs in nanobubbles and conjugation of targeting ligands to develop targeted therapeutic strategies to effect state-specific alteration of the epigenome and hence the disease. To conclude, oxygen nanobubbles can improve cancer outcome through (1) real-time diagnosis and monitoring of tumor hypoxia using ultrasound *in vivo* with high specificity (2) targeted delivery of nanobubbles to specific hypoxic locations within tumors (3) perturbation of tumor’s hypoxia-adaptive pathways and halt in tumor progression and (4) pre-sensitization to epigenetic therapy through 5mC hypomethylation reversal.

## Materials and Methods

### Cell culture

Mouse bladder cancer (MB49) and human cervical cancer, HeLa (ATCC® CCL-2™) cell line was used for *in vitro* and *in vivo* experiments because of their widely studied epigenetic and genome^69^ profiling across various experimental conditions. Cells were cultured in DMEM/F12 media (Gibco, Life Technologies) supplemented with 10% Fetal Bovine Serum (Atlanta Biologicals, Flowery Branch, GA) and 1% Penicillin (10,000 I.U/mL) -Streptomycin (10,000 µg/mL) (Mediatech Inc., Manassas, VA). The cells were routinely cultured at 37 °C in a humidified atmosphere with 5% CO_2_. Cells were tested for mycoplasma contamination using Hoechst 33258 fluorescent indirect staining^70^ before initiating the experiments. Briefly, cells were fixed using 4% paraformaldehyde (PFA) solution and stained with Hoechst 33258 fluorescent dye. Images were obtained using a confocal microscope. No small specks were observed surrounding the cells thus confirming the absence of mycoplasma. Representative images are provided in the supplementary information (Supplementary Fig. 20).

### Synthesis of oxygen nanobubbles

Sodium carboxymethyl cellulose (Aqualon 7HF PH, Ashland Inc., Calumet City, IL) was dissolved in nanopure water to form a 0.1% (w/v) gel and homogenized and saturated with oxygen gas (UHP grade). The oxygen inlet was connected with an air nozzle (Nano Super Air Nozzle 1110SS, EXAIR Corporation) and a 20 nm membrane filter (Emflon II, Pall Corporation) to help generate oxygen nanobubbles. The carboxymethyl cellulose solution was sonicated simultaneously with a probe (Ultrasonic Power Corporation Cell Disrupter) and a bath sonicator (Branson 2210) since ultrasonic energy helps sonic compression of oxygen microbubbles to produce oxygen nanobubbles in the solution^71^. Finally, 1% aluminum chloride (AlCl_3_) cross-linking agent was added to form the encapsulation structure under continuous ultrasonication. Aluminum chloride is a trivalent cross linker and helps decrease the drug release rate^72^ compared to bivalent cross linkers. Aluminum chloride also serves as a strong electrolyte and increases the electrostatic repulsive force to balance out the size reduction forces of the nanobubble, thus stabilizing the nanobubble^73^. The pH of the resulting nanobubble suspension was subsequently neutralized to a pH of 7 using 0.1% ammonium hydroxide (NH_4_OH) solution added drop wise.

To synthesize nanobubble shell (CTRL), the above procedure was repeated without the introduction of oxygen to the polymer solution. The precursor 0.1% (w/v) sodium carboxymethylcellulose solution was bubbled with nitrogen gas before initiating the synthesis of nanobubble shells to remove any pre-dissolved oxygen. The synthesized nanobubble shells were stored in glass vials with the headspace flushed with nitrogen.

### Characterization of Nanobubbles

Transmission electron microscopy (TEM) images of the samples were obtained using a Tecnai T20 transmission electron microscope. Freshly prepared nanobubble solution was dropped directly onto the formvar/carbon 400 mesh TEM grid (Ted Pella Inc., Redding, CA) and air dried for 5 minutes. Further, the sample was negative stained using 4.5% uranyl acetate before being subjected to TEM analyses. Dynamic light scattering (DLS) measurements of the particle hydrodynamic radius were performed using the Zetasizer Nano ZS (Malvern Instruments, Worcestershire, UK) particle size analyzer. Temperature was maintained at 22 °C during analysis and at least 30 measurements were performed on each sample. Data was analyzed with the Zetasizer software.

### Cell count and Cell viability

Cell count and cell viability was measured both before and after the experimental set up initiation and completion respectively. Countess® Automated cell counter (Invitrogen, Life Technologies) was used to count the approximate number of live and dead cells along with the overall cell density in the sample. Dilution of the original cell culture sample by a factor of 2 was carried out in 0.4% trypan blue (Sigma Aldrich). Prepared samples were aliquoted into the counter slides and used for counting.

### Hypoxia Induction

In order to best replicate *in vivo* hypoxic environment and conditions, cells were cultured in 25 cm^3^ flasks at 37 °C in humidified atmospheric conditions with 5% CO_2_ and 2% O_2_. Apart from the standard culture media (described above), no chemical reagents were added to assist hypoxia induction.

### Nanobubble treatment and DNA extraction

Different experimental conditions were utilized to best study the effects on nanobubble concentrations and treatment time on the target cells. The initial setup included a total treatment time of 48 h with nanobubbles (0.5 mg/mL) being added at the start of incubation; after 24 h of incubation; and both at the start and after 24 h of incubation. A negative control was maintained under both normoxic and hypoxic environment without any nanobubble treatment.

We designed and employed an experimental set to measure the concentration dependence of the oxygenated nanobubble treatment and the level of 5mC methylation in HeLa cells cultured under hypoxic conditions. The experimental setup involved the addition of oxygenated nanobubbles (0.1 mg/mL, 0.5 mg/mL, and 1.0 mg/mL) to the experimental cells cultures. The treatment took place every 8 h while the cells were incubated in an incubator kept under hypoxic conditions. After a total incubation time of 48 h, the cell cultures were washed with 1X PBS buffer (Gibco by Life Technologies) and detached from the flask with 0.25% 1X Trypsin-EDTA (Gibco by Life Technologies). DNA was extracted from all the samples using the DNeasy Blood and Tissue Kit (Qiagen). All of the experimental as well as the control cell cultures were run in duplicate to increase the overall accuracy of treatment experiments.

### 5mC methylation quantification by ELISA-based immunoassay

5mC methylation was quantified using protocols published previously^36^. Briefly, all extracted DNA samples were quantified using the NanoDrop ND100 Spectrophotometer. All DNA samples were diluted to 2 ng/µL with DI water. 100 µL of the diluted DNA from each experimental set was added to individual wells in a 96-well plate with 100 µL of Reacti-Bind DNA coating solution (Thermo Scientific). The plate was incubated at room temperature for 4 h on a rocker agitator. After every incubation step, the wells were washed three times with DI water.

After the first round of incubation and washing, 200 µL of 0.5% (w/v) Casein (Sigma Aldrich) prepared in PBS (10 mM PBS with 150 mM NaCl) was added to each well and incubated at 37 °C for an hour. For global assessment of 5mC, 100 µL of 0.5 µg/mL of the primary mouse monoclonal anti 5-Methyl-cytosine (5mC) antibody (Zymo Research Corporation, Clone 10G4, Catalog #A3001-50, Lot # ZRC) was added to each well. Incubation was carried out at 37 °C for 2 h. To tag the primary antibody, 100 µL of 1.0 µg/mL of the secondary antibody, goat anti-mouse IgG-Biotin conjugate (Pierce Thermo Fisher Scientific, Waltham, MA, Catalog # 31802) was added to each well and incubated at 37 °C for 1 hr. To form the HRP-streptavidin conjugate, 100 µL of 0.125 µg/mL of Pierce High sensitivity HRP-labelled streptavidin (Pierce Thermo Fisher Scientific, Waltham, MA) was added to each well and a final incubation was performed at 37 °C for 1 hr. The primary and the secondary antibody along with HRP-streptavidin were diluted in PBS (Life Technologies) containing 0.5% (w/v) Casein (Sigma Aldrich) and 0.1% (v/v) Tween 20 (Bio-Rad). In order to generate color in the assay, each well was treated with 100 µL of 1-Step™ Ultra TMB-ELISA (Pierce Thermo Fisher Scientific, Waltham, MA). After 15 minutes of mild agitation on the rocker, 50 µL of 2 M H_2_SO_4_ was added to each well to stop the color generating reaction. Spectrophotometer readings were taken at 450 nm using the ELISA endpoint model available on the SoftMax Pro 5.2 software pack. Each assay was performed in triplicate to improve the accuracy of measurements.

### Confocal microscopy and imaging

A new culture of HeLa cells was treated with 1.0 mg/mL of oxygen nanobubble (conjugated with Fluorescein isothiocyanate-FitC) solution. After 24 h of incubation, the cells were viewed and imaged under a confocal microscope. Epifluorescence images were obtained with an emission of 488 nm. An Olympus IX71® Inverted Microscope with a × 20 objective lens (Olympus UIS2) was used to view and image the cells. Images were captured with QCapture software and post-processed using ImageJ software.

### *Ex vivo* and cellular ultrasound imaging

Ultrasound imaging was carried out using the Vevo 2100 ultrasound imaging system (FUJIFILM VisualSonics Inc., Toronto, Canada) equipped with a 256-element linear transducer with a center frequency of 40 MHz and a bandwidth of 22–55 MHz (MS550D, Vevo 2100, VisualSonics Inc.). Imaging focal zones, brightness, and contrast were kept constant in all the experiments. Briefly, the center frequency was 40 MHz with a 7mm bandwidth. The imaging plane was 6 mm in depth for the *in vitro* experiments. The transducer tip was immersed 0.5 cm into the water for *ex vivo* bubble imaging (Supplementary Fig. 1). To observe the concentration dependence of ultrasound imaging intensity, different concentrations of nanobubbles were injected into 10 mL DI water placed on top of 5 cm 1% agarose gel phantoms. Images were processed using ImageJ (Research Services, National Institute of Health) software. To obtain *in vitro* ultrasound images, HeLa cells were incubated in 8 cm^2^ (CLS3294- Sigma Aldrich) culture plates with 10 mL culture media for 24 h with and without nanobubbles. Further, the culture plates were imaged using the same ultrasound imaging setup with the transducer tip immersed 0.5 mm into the media. A region of interest was loaded onto each image and the mean grey scale intensity was quantified. The data was exported to JMP software for statistical analysis.

### *In vivo* ultrasound imaging

Male nude BALB/c were used for the study since they are ideal for tumor biology and xenograft research and lack cell-mediated immunity. Mice were anesthetized with 1–2% isoflurane in 1.5 L/min of medical air and nanobubbles (25 µL, 0.5 mg/mL) were administered via subcutaneous or intratumor injection at the right flank. Saline was used as control. Ultrasound imaging was carried out with a 256 element, solid-state 40 MHz transducer. The imaging plane was 10 mm for *in vivo* experiments. Focal planes were set at 4 mm and 6 mm from the transducer surface. Ultrasound gray scale imaging intensity was quantified using ImageJ software. Multiple images of each sample were acquired (n = 5). Further, the grayscale image intensity was quantified using previously established protocol^74, 75^. A selected region of interest was drawn (0.25 mm × 0.25 mm for *in vitro* images; 0.5 mm × 0.5 mm for *in vivo* images) within the sample. For *in vivo* imaging, the tumors were traced approximately in post-processing in ImageJ before quantifying the grayscale intensity inside the boxed region of interest. The region of interest was randomly changed to different locations within the tumor to ensure a good representation of the image. The region of interest selected was significantly smaller than the size of the tumor to ensure that the region does not extend beyond the tumor boundaries. All of the studies were performed with blinding for all researchers collecting the data except the first author PB for whom blinding was not possible. All experiments were performed according to our protocol approved by Purdue Animal Care and Use (PACUC) committee (approval #1404001052).

### *In vivo* imaging and efficacy studies in tumor bearing mice

Animals were cared for under the supervision of the Purdue Animal Care and Use Committee (PACUC). Study was performed with blinding for all researchers collecting data except the first author PB for whom blinding was not possible. MB49 cells (5 × 10^5^ cells/mouse) in the media were subcutaneously injected in female 6–8 weeks old C57Bl/6 mice since C57Bl/6 mice are a syngenic model for MB49 cell line and are immunocompetent. HeLa cells (2 × 10^6^ cells/mouse) suspended in 1:1 media and matrigel were injected subcutaneously in female NOD *scid* gamma (NSG) mice (Jackson Labs Stock #005557, in-house breeding) 20 weeks of age since they are a good model for cancer xenograft models and are the most immunodeficient mice. Number of replicates required for the study was calculated using the power law analysis for *in vitro* and preliminary *in vivo* data using a desired power of test of 90% and alpha of 5%. After ~2 weeks when tumors develop hypoxia and reach an approximate length of 10 mm ± 2 mm, the mice were randomly divided into four groups of five mice each. Tumor-bearing mice were treated by intratumoral injections of oxygen nanobubbles (100 µg/mL, 100 µL, n = 10, 0.1 µg/kg mouse weight), saline (100 µL, n = 10), nanobubble shell (100 µg/mL, 100 µL, n = 5, 0.1 µg/kg mouse weight), or doxorubicin (40 mg/kg mouse weight, 100 µL, n = 10) and given a total of four injections every four days. After dosing, the mice were monitored for weight and implanted tumor size. Oxygen measurements were performed using a localized oxygen probe (Oxylite bare-fiber oxygen sensor, Oxford Optronix, Oxford, England). Representative data is shown for oxygen measurements. Measurements were not repeated further to limit discomfort to mice caused due to the invasive nature of oxygen probe. Further, data obtained *in vivo* followed the trend obtained *in vitro*. Animal euthanasia conditions were followed per the guidelines set by the National Institute of Health (NIH). The criteria for euthanasia was set as 20 mm any dimension of the tumor or any adverse effects or toxicity observed in the mice. The length and width of tumors was measured by tumor calipers and the tumor volume was calculated using the equation:

1 $$Tumor\,volume=\frac{length\times widt{h}^{2}}{2}$$

Tumor volume was also calculated using 3D ultrasound measurements. Four days following bolus injections, mice were euthanized via cardiac puncture and the blood and organs were harvested. Tumor sections were snap-frozen in liquid nitrogen and the blood was stored in lithium-heparin tubes for further analysis.

To measure the effect of oxygen nanobubbles on muscle tissue surrounding the tumor, 100 µL of oxygen nanobubbles (1 mg/mL) was injected intramuscularly in the region around the tumor in MB49 tumor bearing mice (n = 5). B-mode ultrasound images were obtained over a period of 28 days and the tissue dimensions and volume were measured using 3D ultrasound imaging and compared with the images obtained pre-injection.

### DNA extraction from tumors

DNA from tumors harvested from MB49 mice groups was extracted using DNeasy Blood & Tissue kit (Qiagen, Cat. No. 69506) following manufacturer’s protocol. DNA was extracted from harvested HeLa tumors using the DNAzol® Reagent (Life Technologies) kit following manufacturer’s protocol. Briefly, 1 mL of DNAzol was added per 50 mg of tissue and homogenized using a glass homogenizer. The resulting suspension was centrifuged at 12000 × g for 10 minutes followed by the addition of 0.5 mL, 100% ethanol. The extracted DNA was washed and suspended in buffer per manufacturer recommendation.

### Quantitative analysis of 5mC by LC-MS/MS

5mC levels were quantified using LC-MS/MS method developed in our lab at the Bindley Bioscience Center (BBC) and as described previously^7, 36^. Genomic DNA was digested into constituent nucleosides using a nuclease mix (Zymo Research, Irvine, CA) and chromatographic separation of nucleosides was performed using an Agilent 1200 HPLC system with a Waters Atlantis T3 (2.1 × 150 mm, 3.5 µm) reversed phase column, with a flow rate of 0.3 ml/min at ambient temperature. The mobile phase consisted of A (0.1% formic acid in water) and B (0.1% formic acid in acetonitrile), starting with 0% B for 2 minutes, with a linear gradient of 0–10% B from 2–8 minutes, with a linear gradient of 10–60% B from 8–10 minutes, a hold at 60% B from 10–12 minutes. Column re-equilibration was performed of 60–0% B from 12–13 minutes, with a 0% B hold from 13–23 minutes. Online mass spectrometry detection was performed using an Agilent 6460 triple quadrupole mass spectrometer, utilizing positive electrospray ionization mode. The deoxyribonucleosides were evaluated by Multiple Reaction Monitoring using the indicated mass transitions: 228.2 → 112.1 (dC) and 242.2 → 126.1 (5mdC). 5mdC and dC were quantitated with calibration curves generated from authentic standards. Limit of Detection (LOD) for 5mdC (0.09 fmol) was comparable to the LOD obtained by the most sensitive LC-MS/MS method (devised by Chen *et al*.^76^). The calibration curves are provided in Supplementary Figure 22.

### Quantitative real-time PCR

Total RNA was extracted from 30 mg tumor tissue with RNeasy Mini Kit (Qiagen), followed by reverse-transcription with iScript cDNA Synthesis (Bio-Rad). Before cDNA synthesis, genomic DNA was removed with DNase I. PCR reaction was performed in a StepOnePlus system using SYBR Green PCR Master Mix (Life Technologies) and the amplification condition was optimized in the previous study^5^. *ΔΔCt* method was used to normalize the transcription of target genes to the internal control gene GAPDH.

### Gene-specific methylation analysis

DNA from control and nanobubble-treated tumors were extracted with DNeasy Blood & Tissue Kit (Qiagen), according to the manufacturer’s protocol. An EpiTect Methyl II PCR array containing 22 mouse tumor suppressor genes (EAMM-551Z, Qiagen) was used to profile promoter methylation levels. In brief, 250 ng purified DNA was digested with methylation-sensitive endonuclease (unmethylated target promoter will be digested) or methylation-dependent endonuclease (methylated target promoter will be digested). After PCR amplification, the relative level of methylated and unmethylated target promoters was determined.

### Bisulfite pyrosequencing

Bisulfite pyrosequencing was performed by EpigenDx laboratories LLC using their established protocol^59, 77–79^. Briefly, 500ng of extracted DNA was bisulfite treated by EpigenDx using a proprietary bisulfite salt solution. Bisulfite treated DNA was purified using Zymogen DNA columns. For SNP mutation analysis, 5 ng of genomic DNA was used for PCR. The PCR was performed with 0.2 μM of each primer and one of the PCR primers is biotinylated to purify the final PCR product using Sepharose beads. The PCR product was bound to Streptavidin Sepharose HP (GE Healthcare Life Sciences), and purified, washed and denatured using the Pyrosequencing Vacuum Prep Tool (Pyrosequencing, Qiagen). PCR products were sequenced by Pyrosequencing PSQ96 HS System (Pyrosequencing, Qiagen) following manufacturer’s instructions (Pyrosequencing, Qiagen). The methylation status of each locus was analyzed individually as a T/C SNP using QCpG software (Pyrosequencing, Qiagen).

### Histology analysis

Histology slides were prepared at the Purdue Histology and Phenotyping Laboratory (PHPL). Tissues were collected after euthanasia at day 25 for CTRL and ONB shell groups and day 30 for ONB and doxorubicin groups. Harvested tissues and tumors from MB49 syngenic tumor mice were fixed in formalin-free IHC Zinc Fixative (BD Pharmingen), embedded in paraffin, and sectioned into 5 µm slices. Hypoxyprobe™-1 Plus Kit (Hypoxyprobe, Inc, Burlington MA) was used to detect tissue hypoxia in tumors. Mice with established MB49 tumors treated with oxygen nanobubbles or saline (control) were i.v. injected with 60 mg/kg body weight of Hypoxyprobe. After 25 min of labeling, tumors were snap frozen and immunohistochemistry was performed on sectioned tissues per manufacturer’s protocol.

CD31 (ab28364, abcam), Carbonic Anhydrase IX (CAIX, ab184006, abcam), and HIF-1α (ab16066, abcam) immunohistochemistry was performed using buffered zinc formalin fixed tumor sections. Blocking, antigen retrieval, and primary and secondary antibody staining were performed simultaneously for all tissue samples per manufacturer’s protocol by Indiana University School of Medicine.

The tissue sections were scanned using the HistoImage digitization system (Leica Aperio Microsystems Inc.). All of the histology images were analyzed using color deconvolution algorithms in Aperio ImageScope (Leica Biosystems Inc.) or ImageJ (Research Services, National Institute of Health). A representative color deconvolution analysis is provided in Supplementary information Fig. 21. Each tissue section was imaged at × 20 and × 40 magnification. For quantitative analysis, the immunohistochemistry (IHC) Image Analysis Toolbox^80^ (ImageJ, NIH) and Aperio ImageScope color deconvolution algorithm^81^ (Leica Biosystems Inc.) were used. Briefly, color selection is used to select the DAB stained pixels and the background color pixels are eliminated. Further, automatic nuclei segmentation is implemented on the images and positively stained nuclei in DAB stained images are quantified. For CD31, the images were analyzed histomorphometrically^48^. CD31-positive microvessels were counted in random regions of the image at ×40 magnification using ImageJ by analyzing brown-staining endothelial cell or endothelial cell cluster^48, 82^. Data are shown as the number of CD31-positive microvessels/mm^2^ tumor area. Identical threshold limits were utilized for tissue sections from individual markers.

Harvested tissues and tumors from HeLa-xenografted mice were fixed in formalin-free IHC Zinc Fixative (BD Pharmingen), embedded in paraffin, and sectioned into 5 µm slices after hematoxylin and eosin (H&E) staining. Histology slides were imaged under the ×20 and ×40 objective magnification.

### Statistical data analysis

Statistical data analysis was carried using the JMP Statistical Software (SAS Institute Inc., Cary, NC). Sample size was calculated using power law analysis. Assumptions for the tests were checked using normal probability plots and residual plot analysis in JMP software. Two sampled student’s unpaired t-test and Fisher’s least significant difference (LSD) test were used to determine the level of significance at 5% unless mentioned otherwise. Statistical reports including confidence intervals and size effects for all experiments are provided in the supplementary information.

## Electronic supplementary material

Supplementary Information
