# Supplementary material for: Oxygen nanobubbles revert hypoxia by methylation programming
Source: Sci Rep. 2017 Aug 24;7:9268. doi: 10.1038/s41598-017-08988-7 (PMC5570893; doi:10.1038/s41598-017-08988-7)
Supplement: Supplementary file 1 — Supplementary Information [file 41598_2017_8988_MOESM1_ESM.pdf]

Supplementary Information

Oxygen nanobubbles revert hypoxia by methylation programming

Pushpak N. Bhandari[1,4], Yi Cui[1,4], Bennett D. Elzey[2], Craig J. Goergen[3], Christopher M. Long[1,4], and Joseph Irudayaraj[1,4]

[1]Department of Agricultural and Biological Engineering, Bindley Bioscience Center, Purdue Center for Cancer Research, Purdue University, 225 South University Street, West Lafayette, Indiana 47907, USA

[2]Department of Comparative Pathobiology, Purdue University, West Lafayette, Indiana 47907, USA

[3]Weldon School of Biomedical Engineering, Purdue University, West Lafayette, Indiana 47907, USA.

[4]Purdue University Center for Cancer Research, West Lafayette, Indiana 47907, USA.

[*]Corresponding Author

Dr. Joseph Irudayaraj

225 South University Street,

Department of Agricultural and Biological Engineering

Purdue University

West Lafayette, Indiana, 47907

Tel: 765-494-0388

Fax: 765-496-1115

E-mail: josephi@purdue.edu

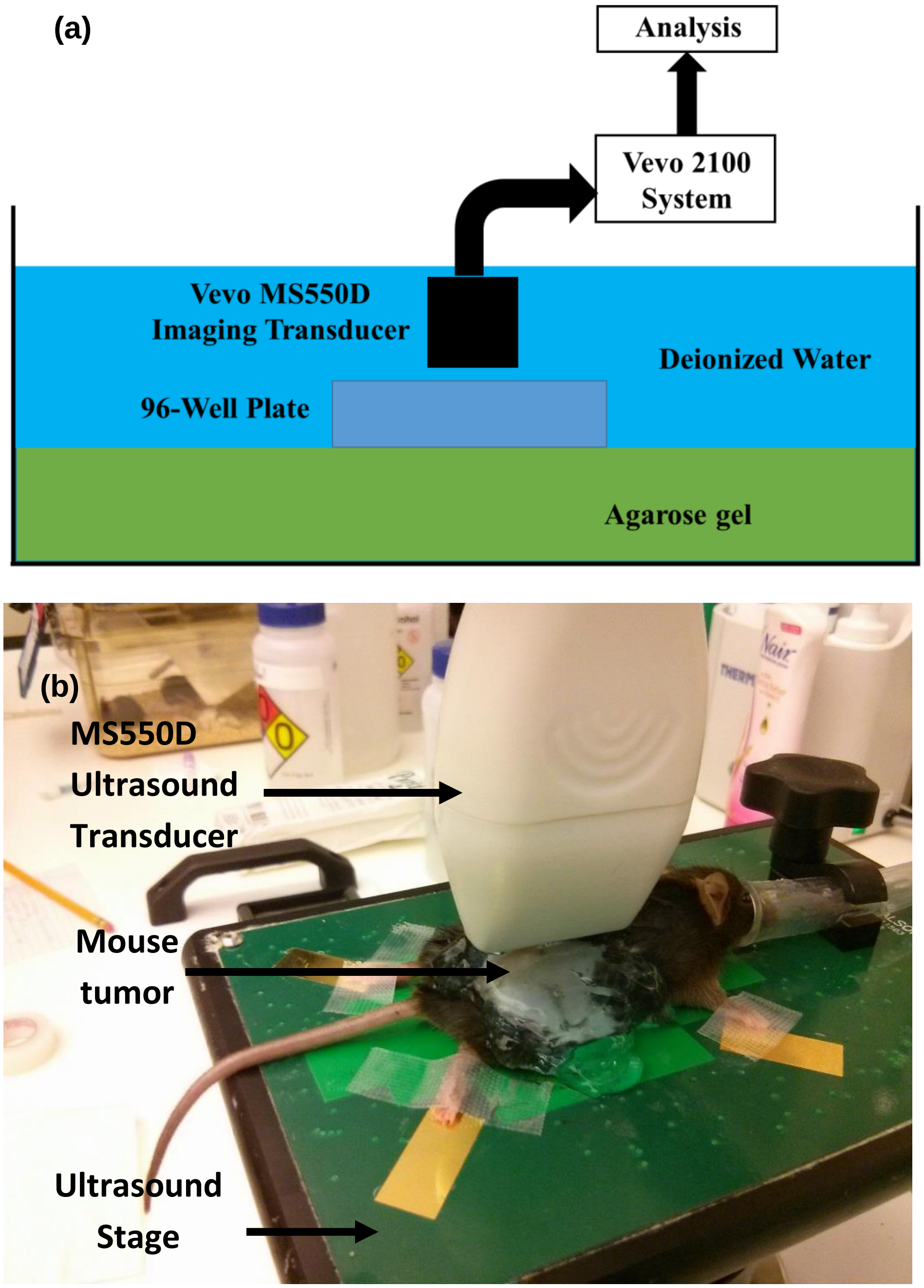

Supplementary Fig. 1: Ultrasound imaging setup for *in vitro* (a) and *in vivo* (b) experiments.

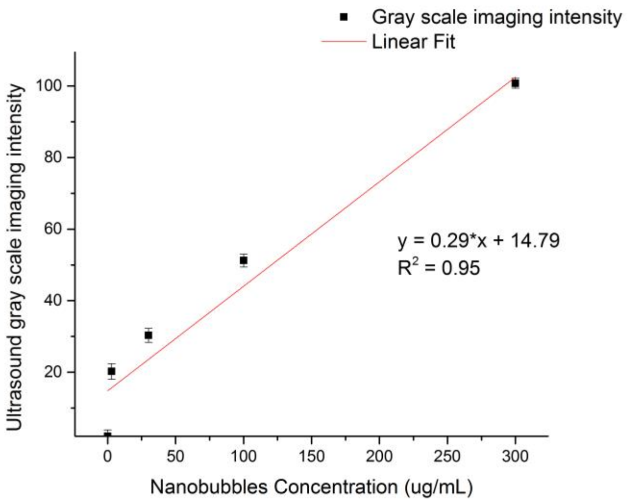

Supplementary Fig. 2: Statistical linear regression analysis for ultrasound gray scale imaging intensity as a function of nanobubble concentration. Results show that the linear model was significant. Linear equation for ultrasound gray scale imaging intensity vs. concentration is: Ultrasound gray scale imaging intensity = 0.29*Nanobubble Concentration (µg/mL) + 14.79. Both, intercept as well as the slope of the equation were significant at $\alpha = 0.05$. $R^2 = 0.95$. The results are mean values from three independent experiments. Error bars represent ± s.d.

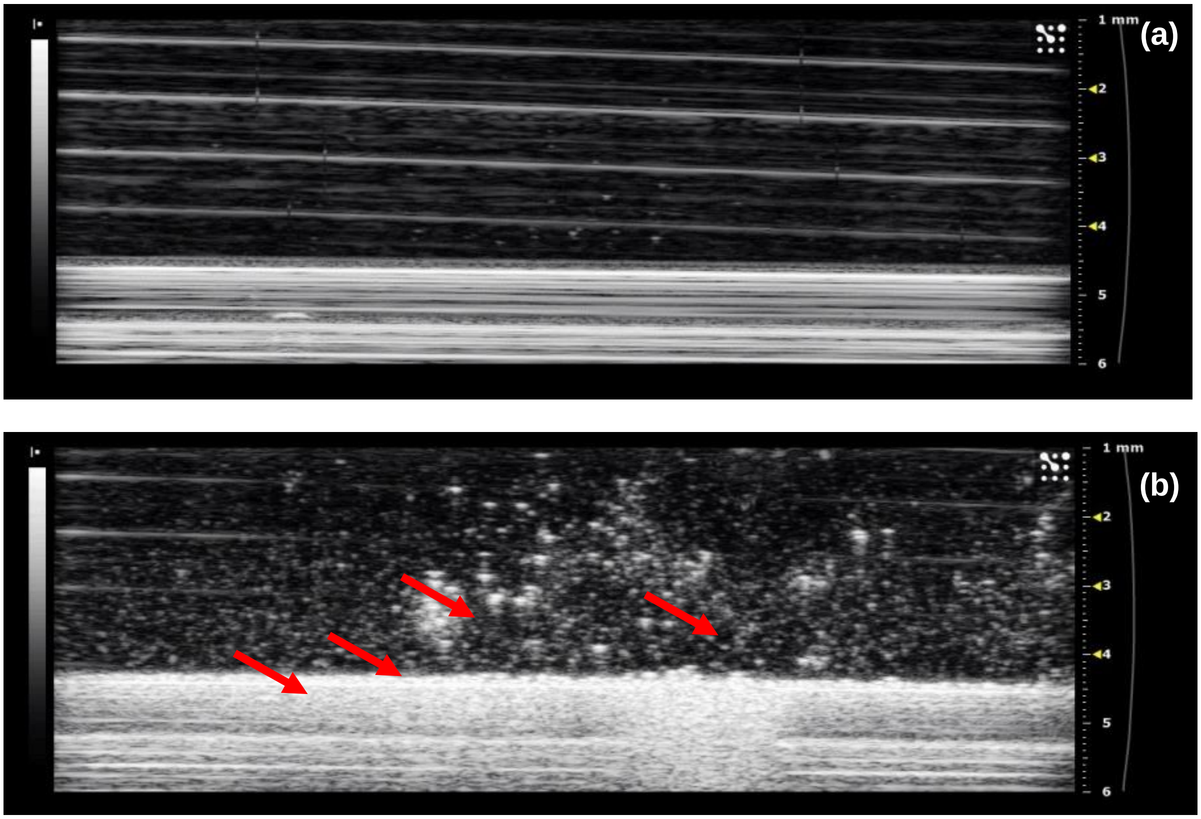

Supplementary Fig. 3: Ultrasound contrast enhancement *in vitro*. (a) Control ultrasound image of HeLa cells on agarose base and media. No nanobubbles were added for this image. **(b)** Ultrasound image to visualize nanobubbles (red arrows). The contrast generated is due to the oxygen gas trapped inside the nanobubbles.

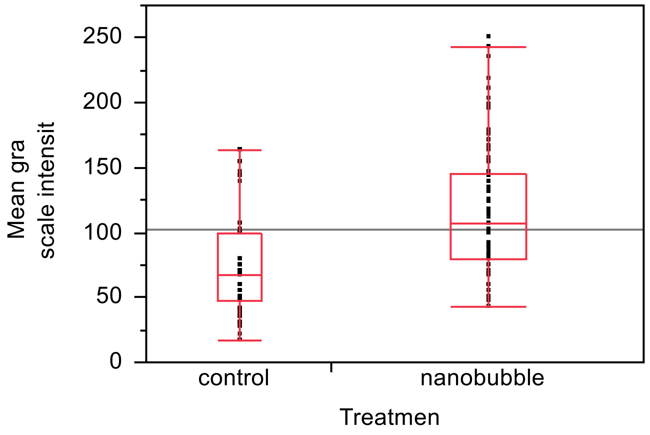

t-Test

nanobubble-control

Assuming equal variances

Difference	37.6248	t Ratio	6.954569
Std Err Dif	5.4101	DF	312
Upper CL Dif	48.2697	Prob > |t|	<.0001*
Lower CL Dif	26.9799	Prob > t	<.0001*
Confidence	0.95	Prob < t	1.0000

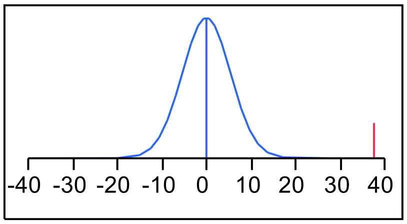

Means and Std Deviations

Level	Number	Mean	Std Dev	Std Err Mean	Lower 95%	Upper 95%
control	117	78.624	42.3778	3.9178	70.86	86.38
nanobubble	197	116.249	48.5506	3.4591	109.43	123.07

Supplementary Fig. 4: Graphical box-plot comparison and statistical one way Student's t-test analysis of ultrasound grey scale intensity of HeLa cells without addition of nanobubbles (control) and with nanobubble treatment (0.5 mg/mL). Ultrasound contrast gray scale imaging intensity was significantly higher ($p < 0.0001$*) for HeLa cells treated with nanobubbles compared to control without nanobubble treatment. Error bars represent ± s.d.

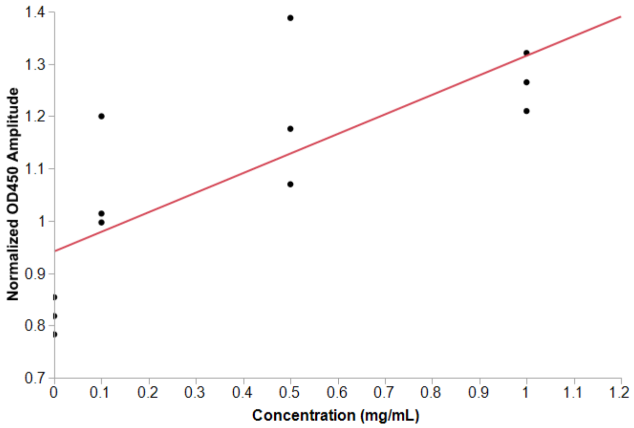

Normalized OD450 Amplitude = 0.9417204 + 0.3740323*Concentration (mg/mL)

Summary of Fit

RSquare	0.584201
RSquare Adj	0.542622
Root Mean Square Error	0.13609
Mean of Response	1.091333
Observations (or Sum Wgts)	12

Analysis of Variance

Source	DF	Sum of Squares	Mean Square	F Ratio
Model	1	0.26021424	0.260214	14.0501
Error	10	0.18520442	0.018520	**Prob > F**
C. Total	11	0.44541867		0.0038*

Parameter Estimates

Term	Estimate	Std Error	t Ratio	Prob>|t|
Intercept	0.9417204	0.056005	16.82	<.0001*
Concentration (mg/mL)	0.3740323	0.099786	3.75	0.0038*

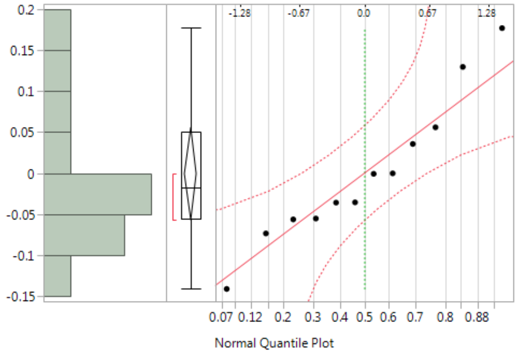

Supplementary Fig. 5: Statistical linear regression analysis for 5mC methylation levels as a function of nanobubble concentration. Results show that the linear model was significant ($p = 0.0038$*). Linear equation for 5mC methylation level vs. concentration is: Normalized OD450 Amplitude = 0.94 + 0.37*Nanobubble Concentration (mg/mL). Both, intercept ($p < 0.0001$*) as well as the slope ($p < 0.003$*) of the equation were significant at $\alpha = 0.05$. $R^2 = 0.58$. The results are mean values from three independent experiments.

Analysis of Variance

Source	DF	Sum of Squares	Mean Square	F Ratio	Prob > F
Column 1	4	1.5917509	0.397938	149.1586	<.0001*
Error	10	0.0266788	0.002668		
C. Total	14	1.6184298			

Connecting Letters Report

Level					Mean
(+/+)	A				1.7618667
(-/+)		B			1.2022667
(+/-)		B			1.1321333
Normoxia control			C		0.9373333
Hypoxia control				D	0.8183333

Levels not connected by same letter are significantly different.

Ordered Differences Report

Level	- Level	Difference	Std Err Dif	Lower CL	Upper CL	p-Value
(+/+)	Hypoxia control	0.9435333	0.0421733	0.849565	1.037501	<.0001*
(+/+)	Normoxia control	0.8245333	0.0421733	0.730565	0.918501	<.0001*
(+/+)	(+/-)	0.6297333	0.0421733	0.535765	0.723701	<.0001*
(+/+)	(-/+)	0.5596000	0.0421733	0.465632	0.653568	<.0001*
(-/+)	Hypoxia control	0.3839333	0.0421733	0.289965	0.477901	<.0001*
(+/-)	Hypoxia control	0.3138000	0.0421733	0.219832	0.407768	<.0001*
(-/+)	Normoxia control	0.2649333	0.0421733	0.170965	0.358901	<.0001*
(+/-)	Normoxia control	0.1948000	0.0421733	0.100832	0.288768	0.0010*
Normoxia control	Hypoxia control	0.1190000	0.0421733	0.025032	0.212968	0.0181*
(-/+)	(+/-)	0.0701333	0.0421733	-0.023835	0.164101	0.1273

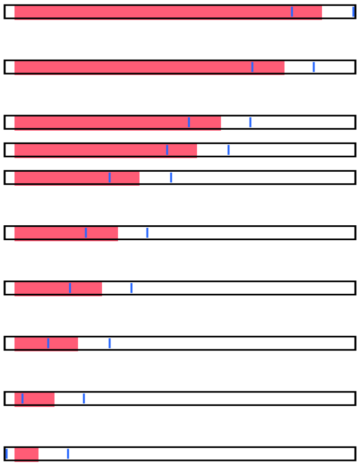

Supplementary Fig. 6: Statistical Analysis of Variance (ANOVA) analysis of 5mC methylation as a function of treatment condition. Results show that ANOVA model was significant ($p < 0.0001$*). The Mean values followed by the same annotation are not significantly different ($\alpha = 5\%$). The results are mean values from three independent experiments.

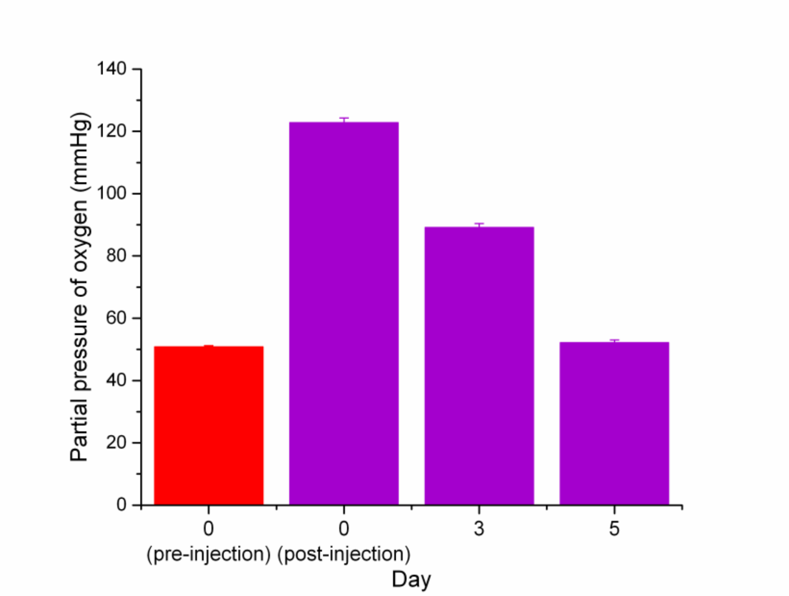

Supplementary Fig. 7: Intratumor partial pressure of oxygen pre-injection and post-injection of ONBs over 5 days. Mice with MB49 tumors were intratumorally injected with ONBs (1 mg/mL ONB, 0.1 µg/kg mouse weight). The oxygen concentrations were measured using OxyLite probe inserted intratumorally. pO_2 readings are averages of recordings made every 60 s for 5 min. (n = 3).

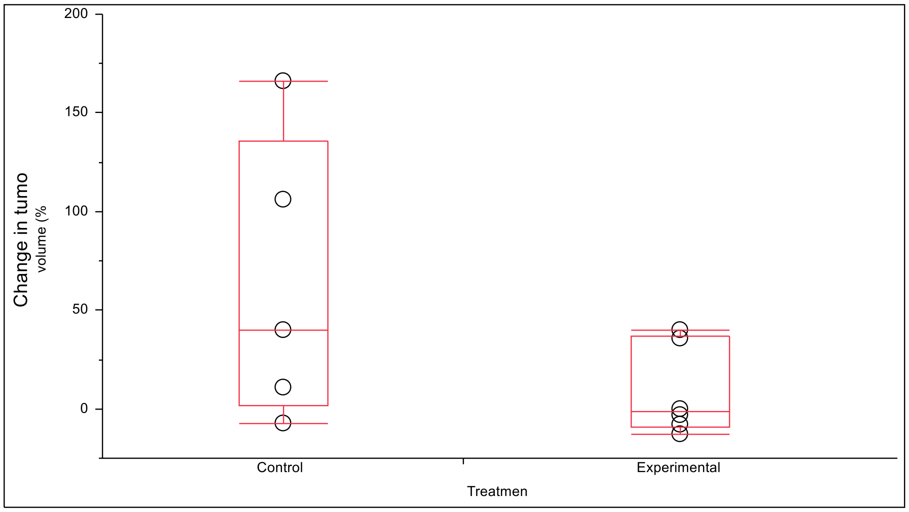

Means and Std Deviations

Level	Number	Mean	Std Dev	Std Err Mean	Lower 95%	Upper 95%
Control	5	62.9799	71.8614	32.137	-26.25	152.21
Experimental	6	8.5367	23.0447	9.408	-15.65	32.72

t Test

Experimental-Control

Assuming unequal variances

Difference	-54.44	t Ratio	-1.62584
Std Err Dif	33.49	DF	4.687417
Upper CL Dif	33.39	Prob > |t|	0.1688
Lower CL Dif	-142.28	Prob > t	0.9156
Confidence	0.95	Prob < t	0.0844

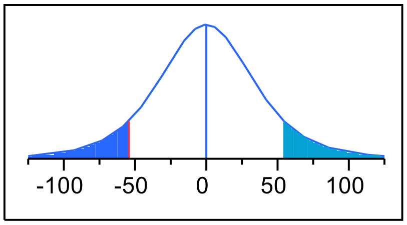

Supplementary Fig. 8: Graphical box-plot comparison and statistical one way ANOVA analysis of change in tumor volume (%) without addition of nanobubbles (control) and with nanobubble treatment (0.5 mg/mL). Change in tumor volume (%) was significantly lower ($p < t = 0.084$) for tumors treated with nanobubbles (n = 6) compared to control without nanobubble treatment (n = 5). Error bars represent ± s.d.

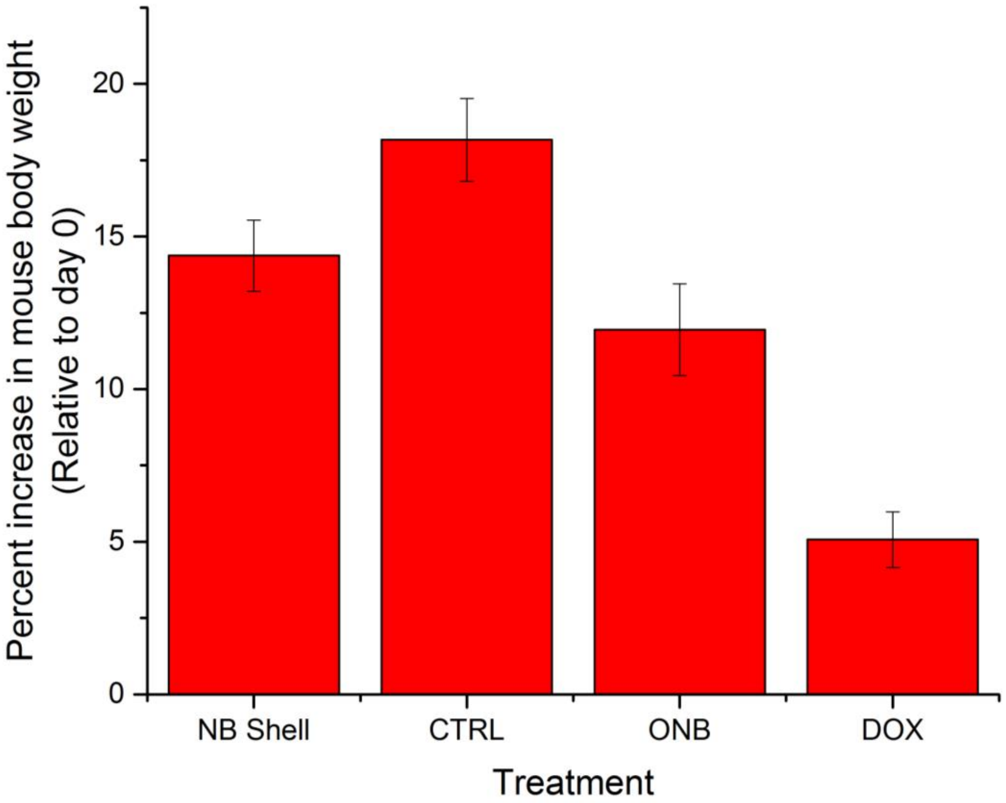

Supplementary Fig. 9: Toxicity of various treatments in tumor bearing mice. Percent change in mouse weight showed significant toxicity induced by doxorubicin treatment on mouse weight compared to ONB.

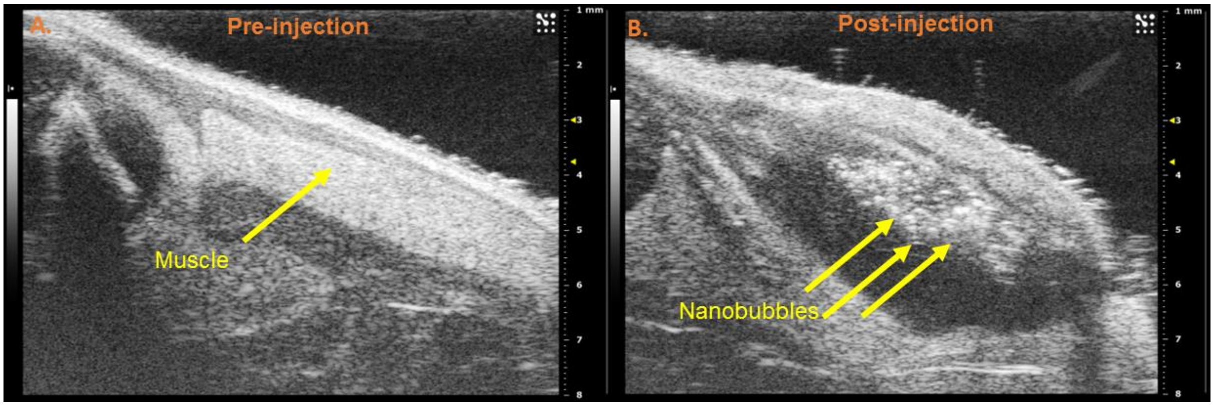

Supplementary Fig. 10: Nanobubbles specificity to hypoxic tumor *in vivo*. (a) Representative ultrasound B-mode image of muscle tissue surrounding tumor before injection and **(b)** post-injection of nanobubbles. No significant growth/necrosis was observed in the normal adjacent muscle tissue for the duration monitored (28 days) highlighting specificity of nanobubbles towards hypoxic tumors.

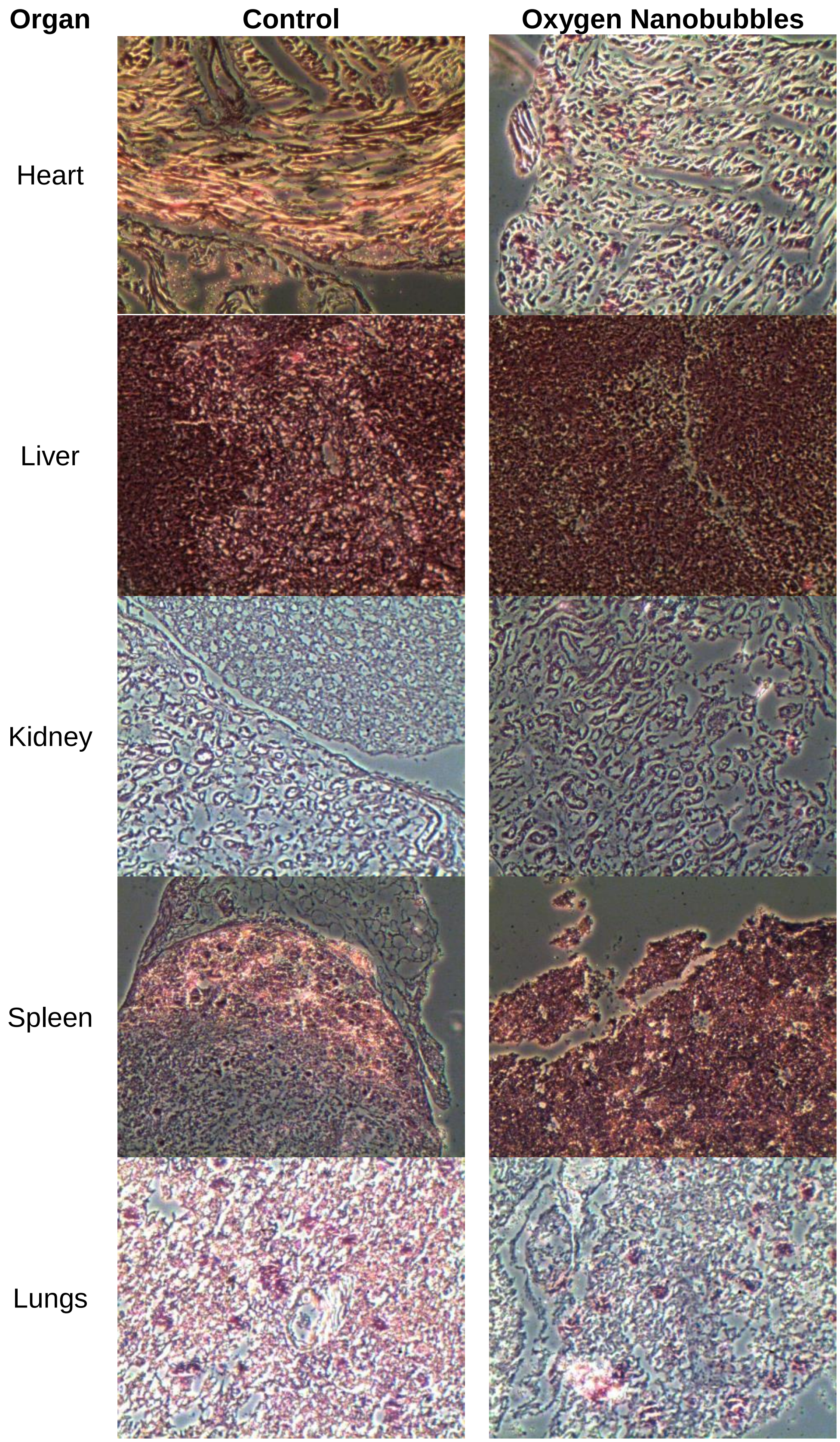
Organ
Control
Oxygen Nanobubbles
Heart
Liver
Kidney
Spleen
Lungs

Supplementary Fig. 11: Biocompatibility of nanobubbles. Heart, liver, kidney, spleen, and lung histology were conducted. Organs were collected from mice at the end of the study and stained with haematoxylin and eosin. No noticeable lesions or tissue damage were observed in any of the organs treated.

HIF-1α	(F) GAAAGCGCAAGTCTTCAAAG<br>(R) TGGGTAGGAGATGGAGATGC
PDK1a	(F) CCAGTGGATAAGCGGAAGGG<br>(R) CCTGTTAGGCGTGTGGACAA
MAT2A	(F) ATGAACGGACAGCTCAACGG<br>(R) CCAGCAAGAAGGATCATTCCAG

Supplementary Fig. 12: Table indicating PCR primers used for HeLa xenograft tumors.

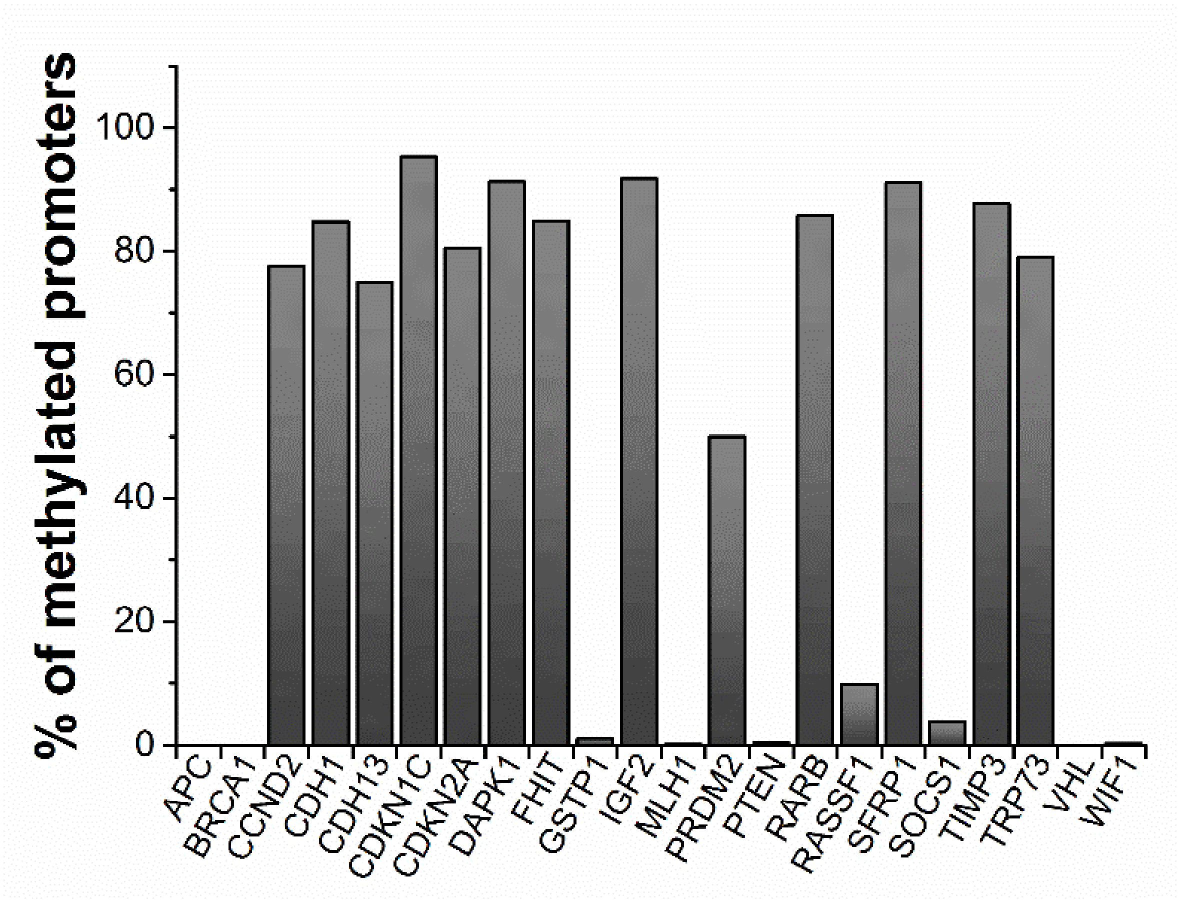

Supplementary Fig. 13: The original methylation level of 22 tumor suppressor genes.

	Apoptosis	Extracellular Matrix & Adhesion	Cell Cycle	DNA Damage & Repair	Signal Transduction	Transcription Factors
**APC**		√	√	√	√	
**BRCA1**	√		√	√	√	√
**CCND2**			√			
**CDH1**		√				√
**CDH13**		√			√	
**CDKN1C**			√			
**CDKN2A**	√	√	√			
**DAPK1**	√					
**FHIT**			√			
**GSTP1**	√					
**IGF2**					√	
**MLH1**			√	√		
**PRDM2**						√
**PTEN**	√		√		√	
**RARB**						√
**RASSF1**			√		√	
**SFRP1**		√			√	
**SOCS1**					√	
**TIMP3**	√					
**TRP73**	√		√	√		√
**VHL**	√		√			√
**WIF1**					√	

Supplementary Fig. 14: Function and pathways involving the assessed 22 tumor suppressor genes.

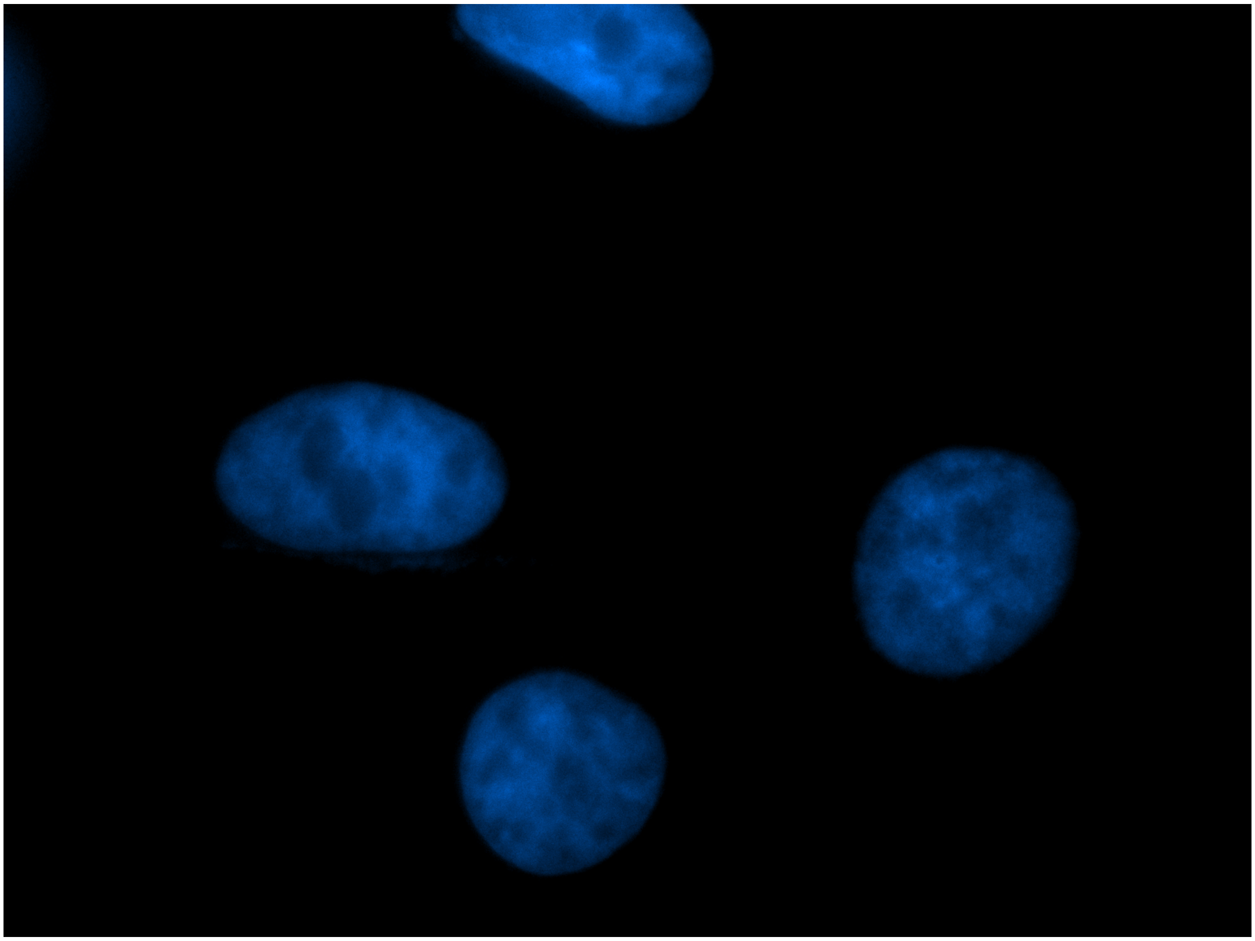

Supplementary Fig. 15: Mycoplasma contamination test. Mycoplasma contamination was tested using indirect fluorescent staining with Hoechst 33258 dye. No specks or small dots of fluorescence surrounding the cell surface or in the surrounding medium in images obtained using confocal microscope confirmed the absence of mycoplasma contamination. Representative image obtained before initializing the experiments is shown.

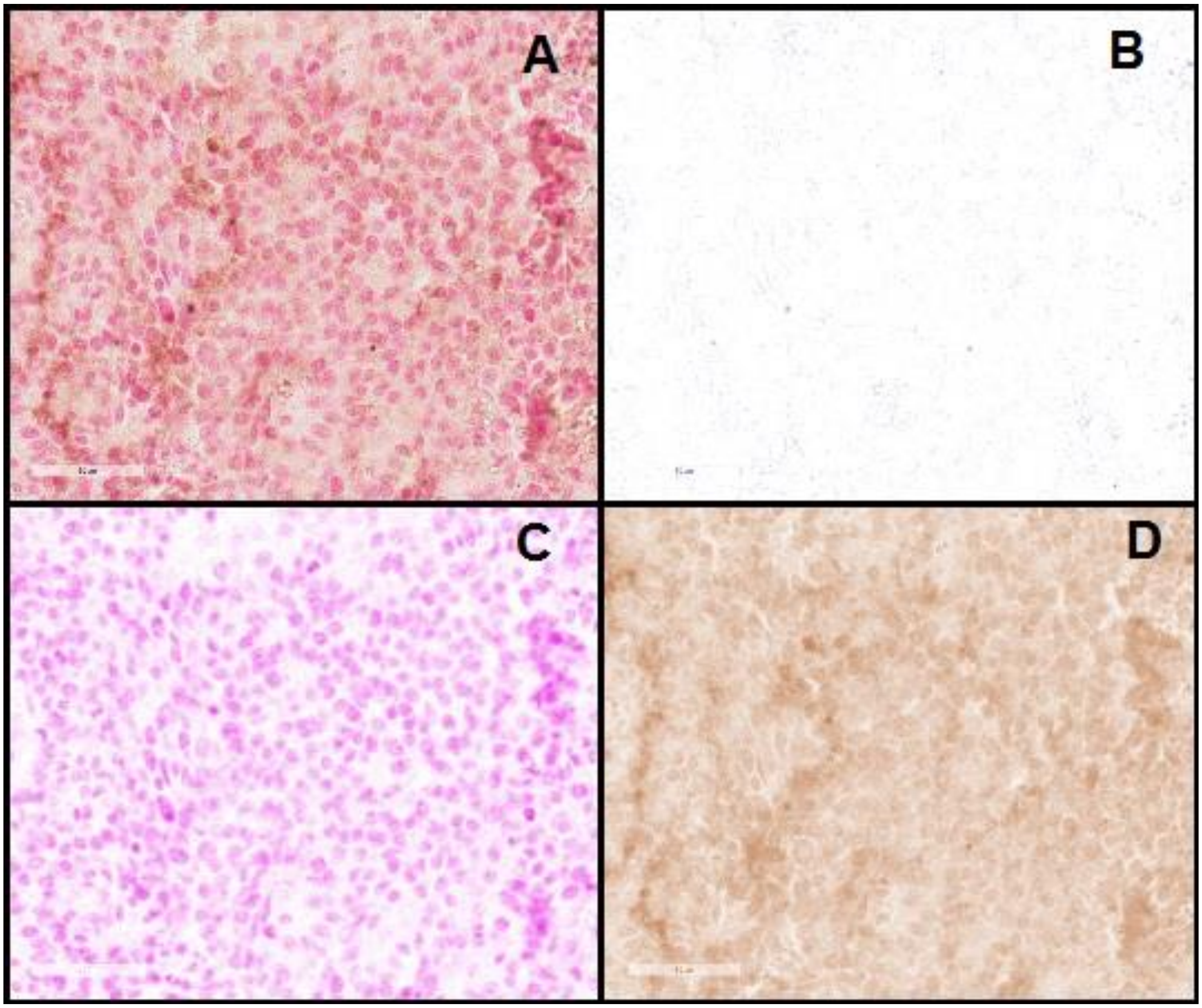

Supplementary Fig. 16: Representative color deconvolution analysis result for HIF-1α immunohistochemistry. Color selection is used to select the DAB stained pixels and the background color pixels are eliminated. Further, automatic nuclei segmentation is implemented on the images and positively stained nuclei in DAB stained images are quantified. (A) Representative IHC tissue image stained for HIF-1; 8-bit image showing background stain; (C) Eosin-only-image containing pink eosin-stained cells; and (D) DAB-only-image.

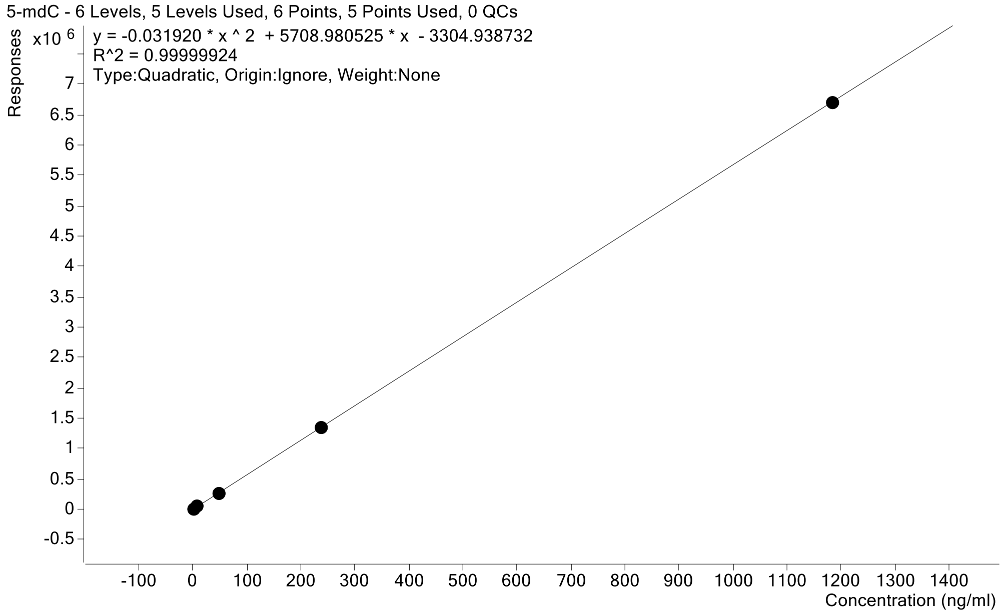

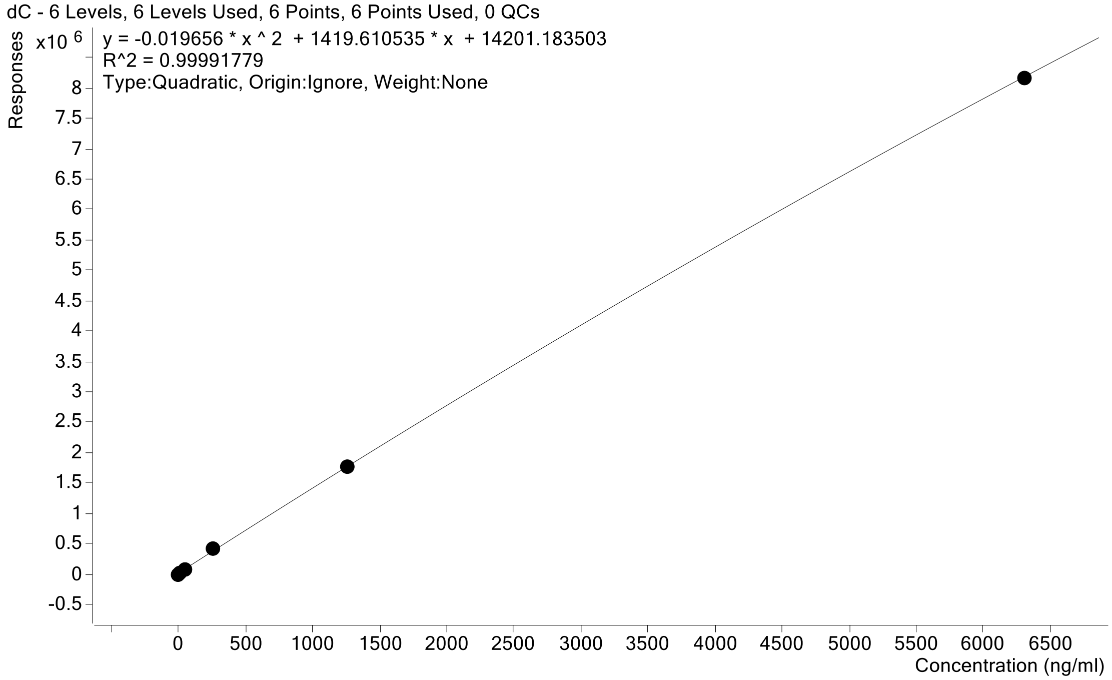

Supplementary Fig. 17: 5mdC **(a)** and dC **(b)** calibration curves generated using LC-MS/MS. Calibration was made using a 6-point curve.
